# Facilitators and barriers of preventive behaviors against COVID-19 during Ramadan: A phenomenology of Indonesian adults

**DOI:** 10.3389/fpubh.2023.960500

**Published:** 2023-03-21

**Authors:** Rakhmat Ari Wibowo, Romi Bhakti Hartarto, Arnab Bhattacharjee, Dyah Titis Kusuma Wardani, Novat Pugo Sambodo, Prattama Santoso Utomo, Luthvia Annisa, Mohamad Saifudin Hakim, Meida Sofyana, Fatwa Sari Tetra Dewi

**Affiliations:** ^1^Department of Physiology, Faculty of Medicine, Public Health and Nursing, Universitas Gadjah Mada, Yogyakarta, Indonesia; ^2^Department of Economics, Faculty of Economics and Business, Universitas Muhammadiyah Yogyakarta, Yogyakarta, Indonesia; ^3^Edinburgh Business School, Heriot-Watt University, Edinburgh, United Kingdom; ^4^The National Institute of Economic and Social Research, London, United Kingdom; ^5^Center for Health Financing Policy and Health Insurance Management, Faculty of Medicine, Public Health and Nursing, Universitas Gadjah Mada, Yogyakarta, Indonesia; ^6^Department of Economics, Faculty of Economics and Business, Universitas Gadjah Mada, Yogyakarta, Indonesia; ^7^Department of Medical Education and Bioethics, Faculty of Medicine, Public Health and Nursing, Universitas Gadjah Mada, Yogyakarta, Indonesia; ^8^Department of Microbiology, Faculty of Medicine, Public Health and Nursing, Universitas Gadjah Mada, Yogyakarta, Indonesia; ^9^Department of Health Behavior, Environment and Social Medicine, Faculty of Medicine, Public Health and Nursing, Universitas Gadjah Mada, Yogyakarta, Indonesia

**Keywords:** COVID-19, preventive behavior, mobility restriction, physical distancing, face mask

## Abstract

**Introduction:**

Intercity mobility restriction, physical distancing, and mask-wearing are preventive behaviors to reduce the transmission of COVID-19. However, strong cultural and religious traditions become particular challenges in Indonesia. This study uses the Behavior Change Wheel to explore barriers and facilitators for intercity mobility restriction, physical distancing, and mask-wearing during Ramadan.

**Methods:**

Semi-structured in-depth interviews with 50 Indonesian adults were conducted between 10 April and 4 June 2020. Having mapped codes into the Capacity, Opportunity, Motivation – Behavior (COM-B), and Theoretical Domain Framework (TDF) model, we conducted summative content analysis to analyze the most identified factors to preventive behaviors and proposed interventions to address those factors.

**Results:**

Belief about the consequence of preventive behaviors was the most mentioned facilitator to all preventive behaviors among compliers. However, optimism as a TDF factor was commonly mentioned as a barrier to preventive behaviors among non-compliers, while environmental context and resources were the most commonly mentioned factors for intercity mobility restriction.

**Conclusions:**

Public health intervention should be implemented considering the persuasion and involvement of religious and local leaders. Concerning job and economic context, policy related to the intercity mobility restriction should be reconsidered to prevent a counterproductive effect.

## 1. Introduction

The occurrence of novel coronavirus disease 2019 (COVID-19) caused by infection of SARS-CoV-2 viruses has spread dramatically worldwide since its first cluster of cases was reported in December 2019 in Wuhan city, Hubei Province, China. It is a highly transmissible disease that is primarily transmitted via droplets from sneezing or coughing and via fomites and airborne aerosols in certain situations (1–6). Due to the high transmission of COVID-19 to many countries with a continuous and high rise in morbidity and mortality, the WHO has declared a public health emergency since January 30, 2020. Given that there was no vaccine or effective pharmaceutical treatment at that time, behavioral interventions promoting hand washing, physical distancing, and wearing a face mask were recommended to reduce the SARS-CoV-2 transmission (7, 8). In addition, international public health officials have proposed several cordon-sanitaire measures to mitigate the virus transmission, such as lockdowns, strict quarantine measures, and restraining flights from and to infected countries (9, 10). However, it took many more weeks for many countries to implement precautionary measures, including Indonesia.

Official data report that the severity of COVID-19 has been less alarming in Indonesia than that in many other countries. The Indonesian Government was at first condemned for not responding promptly and obviously to the emergence of the COVID-19 outbreak (11–13). When two neighboring countries, Malaysia and Singapore, observed sharp growth in the COVID-19 spread, Indonesia claimed to have zero cases throughout February 2020 (14). Only on March 2, 2020, the first two confirmed cases were reported in Indonesia. The Indonesian Government ruled out lockdowns, highlighting their severe economic impact in India. The Government then opted to promote some preventive behaviors to reduce the transmission of COVID-19, such as frequent handwashing with soap or hand sanitizer, wearing a face mask, and applying physical distancing. People were also encouraged to stay at home and work from home to reduce physical contact between infected and uninfected individuals. While the health promotion successfully increased public handwashing practice, public compliance with physical distancing and wearing a face mask was below the required level for curtailing the COVID-19 burden (15, 16). In the absence of stringent preventive measures, the confirmed cases of COVID-19 were subsequently found in all of Indonesia's 34 provinces only a month after the first reported case.

The Indonesian Government's efforts to curb the spread of COVID-19 became increasingly challenging, especially when it came to Ramadan and Eid Al-Fitr in May 2020. As a country with the largest Muslim population, Indonesia has some traditions during Ramadan and Eid Al-Fitr, such as homecoming, Ramadan dinner gathering, congregational night prayer, congregational Eid prayer, visiting cemeteries, and *halal bi halal* (asking forgiveness from one another by handshaking). All these traditions bring many people to the same place simultaneously, which consequently presents certain challenges to the efforts toward COVID-19 prevention in Indonesia.

To deter further transmission of COVID-19 to other regions, the Government decided to implement large-scale mobility restrictions by imposing a ban on homecoming activities 1 month before Eid Al-Fitr through Regulation of the Minister of Transportation No. 25 of 2020. Along with the two of the most prominent Islamic organizations (Muhammadiyah and Nahdlatul Ulama), The Government also suggested that all Muslims pray Eid at home rather than in the mosque or open space and encourage the public to comply with the preventive behaviors during the practice of cultural and religious tradition, such as allowing stretching out the prayer rows and wearing a face mask in congregational prayer during the pandemic situation. However, the COVID-19 National Task Force revealed that people neglected preventive measures and celebrated cultural and religious traditions, as usual, resulting in a spike in COVID-19 cases in Indonesia after Eid Al-Fitr (17, 18). The public adherence to preventive behaviors dropped further until the Government implemented a policy involving the local community, called micro-scale restrictions which were imposed on public activities. The situation is still relevant nowadays, especially when adherence to preventive behaviors continuously declined since the new year 2022 (19), followed by a dramatic upsurge of new daily cases caused by the omicron variants (20).

The present study explores how individuals respond to three precautionary measures of COVID-19 during the annual Ramadan month, including travel restrictions, physical distancing, and wearing face masks. Identifying what facilitates and obstructs compliance with preventive measures is pivotal to informing public health interventions and policies. Designing interventions and policies based on theoretical frameworks have been suggested to improve effectiveness (21). The Behavior Change Wheel (BCW) provides comprehensive tools for designing behavioral interventions (22). Assessing factors that influence behavior helps tailor behavior interventions (23, 24). The Capability, Opportunity, Motivation – Behavior (COM-B) model in the center of the BCW is adopted to identify facilitators and barriers of several health behaviors as a basis for designing behavioral interventions (25–30).

Different from the existing literature (31–33), this study examined preventive behaviors in a developing country with strong tradition and cultural factors in a particular annual religious event. The present study uses the COM-B model to explore the barriers and enablers of intercity mobility restriction, physical distancing, and wearing a mask and discuss the proposed interventions and policies to promote these preventive behaviors.

## 2. Methods

### 2.1. Study design

This qualitative research applied a phenomenology approach. Semi-structured interviews with Indonesian adults were conducted from 10 April to 4 June 2020. The phenomenology design was used to investigate individual experiences on COVID-19 preventive behaviors during Ramadan. The design aimed to enhance understanding of the various responses and perspectives on the particular phenomenon (34). This study has obtained ethical approval from the Medical and Health Research Ethics Committee of the Faculty of Medicine, Public Health and Nursing, Universitas Gadjah Mada No: KE-FK-0788-EC-2020 and Social Sciences Ethics Committee of Heriot-Watt University No. 2020-0433-1353. We reported our findings following the Standards for Reporting Qualitative Research (SRQR) checklist (35).

### 2.2. Informants and data collections

A purposive sampling technique was performed by five field epidemiologists representing five regions in Java to endeavor a maximum sample variation (i.e., age, work type and status, religion, ethnicity, income level, comorbidity, and region of domicile), hence opinions could be collected from multiple perspectives following the BCW framework (22, 36). Maximum variation of the sample might ensure the adequacy and authenticity of the obtained data (34). Participation in the interviews was voluntary so long as the informants met inclusion criteria such as being 18 years or older, willing to be contacted by phone for an interview, and living in Greater Jakarta, West Java, Central Java, Yogyakarta, or East Java.

The selection of the above areas is based on most of Indonesia's confirmed cases coming from Java, the most densely populated island in the country (37). The capital city of Jakarta was the epicenter of the pandemic, contributing half of the total positive cases in Indonesia during the first month, and even two-thirds if involving adjacent urban districts that constitute Greater Jakarta (38). Following Jakarta, West Java, Yogyakarta, Central Java, and East Java are other provinces in Java with high numbers of confirmed daily cases.

Study informants were purposively selected based on a database provided by the represented epidemiologist in each district using a quota system based on age, gender, work type and status, income level, comorbidity, and domicile. This study aimed to account for the heterogeneity of the informants about adherence to mobility restriction, physical distancing and wearing a face mask. All interviews used the local language (Java and Madura) and Bahasa Indonesia and were conducted by experienced interviewers who were familiar with the research topic and did not have any personal relationship with informants. Written consent was provided in advance of the interview, and verbal consent was carried out before commencing the interview. We started by interviewing ten informants, and various themes emerged in response to the interview questions. We stopped recruiting informants for interviews if we approached data saturation from recurring responses, i.e., additional informants did not reveal new themes (39). This resulted in 50 informants being interviewed, while interviews lasted between 40 and 60 min.

Semi-structured in-depth interviews were conducted since they were regarded as most appropriate for exploring informants' capacity, opportunity, and motivation to comply or not comply with mobility restrictions and other preventive measures. The semi-structured interview was chosen to ensure informants could express their thoughts without influence from others so the interview could capture more details than the focus group discussion (40, 41). Due to mobility restrictions and physical distancing measures during unusual conditions, the survey was carried out *via* phone interviews to prevent virus transmission.

The interview schedule consisting of open-ended questions and prompts was developed to explore informants' demographic information, general knowledge, perceived compliance to preventive behaviors, and perceptions of enablers and barriers to preventive behaviors. Additional probes and prompts were added based on new topics introduced by informants (42). Two epidemiology experts evaluated the relevance of the survey questions, and the interview guide was modified as suggested.

### 2.3. Analysis

The phone interviews were audio-recorded and transcribed verbatim into Bahasa Indonesia, then translated into English. Transcripts were iteratively coded using inductive thematic analysis to identify common perceptions and opinions, following the analysis process by Braun and Clarke (43). Audio recording and transcript files were stored in encrypted cloud storage. First, we assigned pseudonyms to protect the identities of informants. Then, RAW, a public health researcher, and RBH, a social science researcher, analyzed the interview transcripts independently to allow researcher triangulation. Each of them looked through the transcripts carefully and then generated label codes. Each code was noted as either “facilitators” or “barriers”, depending on the context of the code captured from the informant. Each researcher also kept a reflective note to ensure an obvious coding process. The following process included comparing and cross-checking the coding by two researchers to agree upon common codes. Discrepancies in codes were discussed and resolved with the research team until a consensus was reached. This process ensured credibility and trustworthiness since no key themes were missed. Selected quotes from the informants about what would facilitate or hinder compliance toward mobility restriction and other preventive measures are presented in the results section.

Having reached an agreement on the codes, RAW and RBH independently categorized each code into fourteen domains using the Theoretical Domain Framework (TDF) and mapped it onto the COM-B components (22, 44). Discussion between researchers was conducted to resolve any difference in the TDF and COM-B model code mapping. After agreement on TDF and COM-B mapping had been made, RAW conducted the summative content analysis by discovering the occurrences of codes and calculating the frequency count of coding for each TDF and COM-B domain (45). RAW then made a rank-ordered TDF and COM-B domain according to the frequency coding to point out which TDF and COM-B components were the main facilitators and barriers to compliance with a mobility restriction, physical distancing, and wearing a mask behavior.

The COM-B model has been applied in various contexts (46–48). This model is introduced by Michie et al. (22), positing that people need capability (C), opportunity (O), and motivation (M) to effectuate a behavior (B). This model aims to guide understanding of behavior and develop behavioral targets to be a foundation for intervention design. The model suggests that for individuals to engage in a specific behavior (B), they must be psychologically and physically able (C) and supported by physical and social opportunity (O) to execute the behavior, while the motivation encompasses basic drivers such as habit and impulses (automatic process) as well as intention and choice (reflective process). The COM-B model can be elaborated with more details by the TDF consisting of fourteen domains, allowing researchers to analyze the most important domain-specific aspects related to the target behavior (49–51). Analyzing the factors influencing the behavior will help design interventions based on nine intervention functions and seven policy categories (22).

## 3. Results

A total of fifty informants were interviewed (27 males, and 23 females). The percentage of informants who complied with the mobility restriction, physical distancing, and wearing a face mask behavior during the survey period was 40, 32, and 64%, respectively. Table 1 presents the demographic and other characteristics of informants.

**Table 1:** Characteristics of the participants.

Gender	Male	27
	Female	23
Age	>45	10
	≤ 45	40
Essential work	Yes	16
	No	34
Monthly income	Yes	33
	No	17
Above regional income	Yes	24
	No	14
	Prefer not to say	12
Presence of comorbid	Yes	47
	No	3
Level of education	Elementary degree	1
	High school degree	10
	College degree	20
	Prefer not to say	19
Domicile	Greater Jakarta	10
	West Java and Banten	10
	Central Java	10
	Yogyakarta	10
	East Java and Madura	10
Perceived their self as compliers to a mobility restriction	Yes	20
	No	30
Perceived their self as compliers to physical distancing	Yes	16
	No	34
Perceived their self as compliers to wearing a face mask	Yes	34
	No	16

### 3.1. Behavior analysis using the TDF and COM-B model

Table 2 reports the COM-B components' frequency that influences the intercity mobility restriction, physical distancing, and face mask-wearing behavior. In total, factors that influence physical distancing were mentioned 336 times, making this behavior have the highest number of mentioned factors while wearing a face mask has the least number of mentioned factors. There are some differences in the pattern of COM-B components as mentioned by compilers and non-compliers and the pattern of factors that influence each behavior. Among compliers, they mentioned more facilitators than barriers. In contrast, non-compliers mentioned more barriers than facilitators, except for wearing a face mask. Motivation was the most frequently mentioned facilitator of preventive behavior, except for the intercity mobility restriction since the most frequently mentioned facilitator among non-compliers was the opportunity.

**Table 2:** Summary of COM-B components.

**Behavior**	**Compliance**	**Barriers**			**Facilitators**		
		**COM-B**	**Frequency of mentions**	**Percentage of mentions**	**COM-B**	**Frequency of mentions**	**Percentage of mentions**
Mobility restriction	Compliers	Motivation	6	75%	Motivation	27	46%
		Opportunity	2	100%	Opportunity	26	44%
		Capability	0	0%	Capability	6	10%
	Total mentions	8			59		
	Non-compliers	Opportunity	70	63%	Opportunity	31	54%
		Motivation	42	37%	Motivation	24	42%
		Capability	3	3%	Capability	2	4%
	Total mentions	115			57		
Physical distancing	Compliers	Opportunity	18	75%	Motivation	33	40%
		Motivation	6	25%	Opportunity	28	34%
		Capability	0	0%	Capability	22	27%
	Total mentions	24			83		
	Non-compliers	Opportunity	66	52%	Motivation	45	48%
		Motivation	60	44%	Opportunity	38	40%
		Capability	9	7%	Capability	11	12%
	Total mentions	135			94		
Wearing a face mask	Compliers	Capability	29	88%	Motivation	77	53%
		Opportunity	4	11%	Opportunity	37	26%
		Motivation	2	6%	Capability	31	21%
	Total mentions	35			145		
	Non-compliers	Capability	20	59%	Motivation	29	67%
		Motivation	14	35%	Opportunity	12	28%
		Opportunity	6	15%	Capability	2	5%
	Total mentions	40			43		

The most commonly reported TDF domains that hinder the intercity mobility restriction were almost similar between compliers and non-compliers, including emotion, environmental context and resource, and social influence (Supplemental File 1). While the environmental context and resource domain was the most mentioned facilitator to the intercity mobility restriction among both compilers and non-compliers, this domain was also the most mentioned barrier to the physical distancing behavior. Belief about consequence was found in the top three mentioned facilitators to the intercity mobility restriction and physical distancing among compliers. However, this domain was not found in the top three mentioned facilitators to the other two preventive behaviors among non-compliers.

On the other hand, optimism was among the most commonly mentioned barriers to applying physical distancing and wearing a face mask among non-compliers. However, this domain was not found in the most commonly mentioned barriers to these two-preventive behaviors among compliers. Physical skill as the most mentioned barriers to wearing a face mask, emotion and belief about consequence as the most commonly mentioned facilitators to wearing a face mask were found among both compilers and non-compliers of this behavior. Meanwhile, memory was only mentioned in wearing a face mask behavior.

In total, the determinants of physical distancing were categorized into 56 sub-themes as preventive behavior with the most varied sub-themes compared to those of other preventive behaviors (Supplemental File 2). Intercity mobility restriction and wearing a face mask behavior only had 45, and 44 mentioned influencing factors, respectively.

### 3.2. Intercity mobility restriction

**Figure 1:**
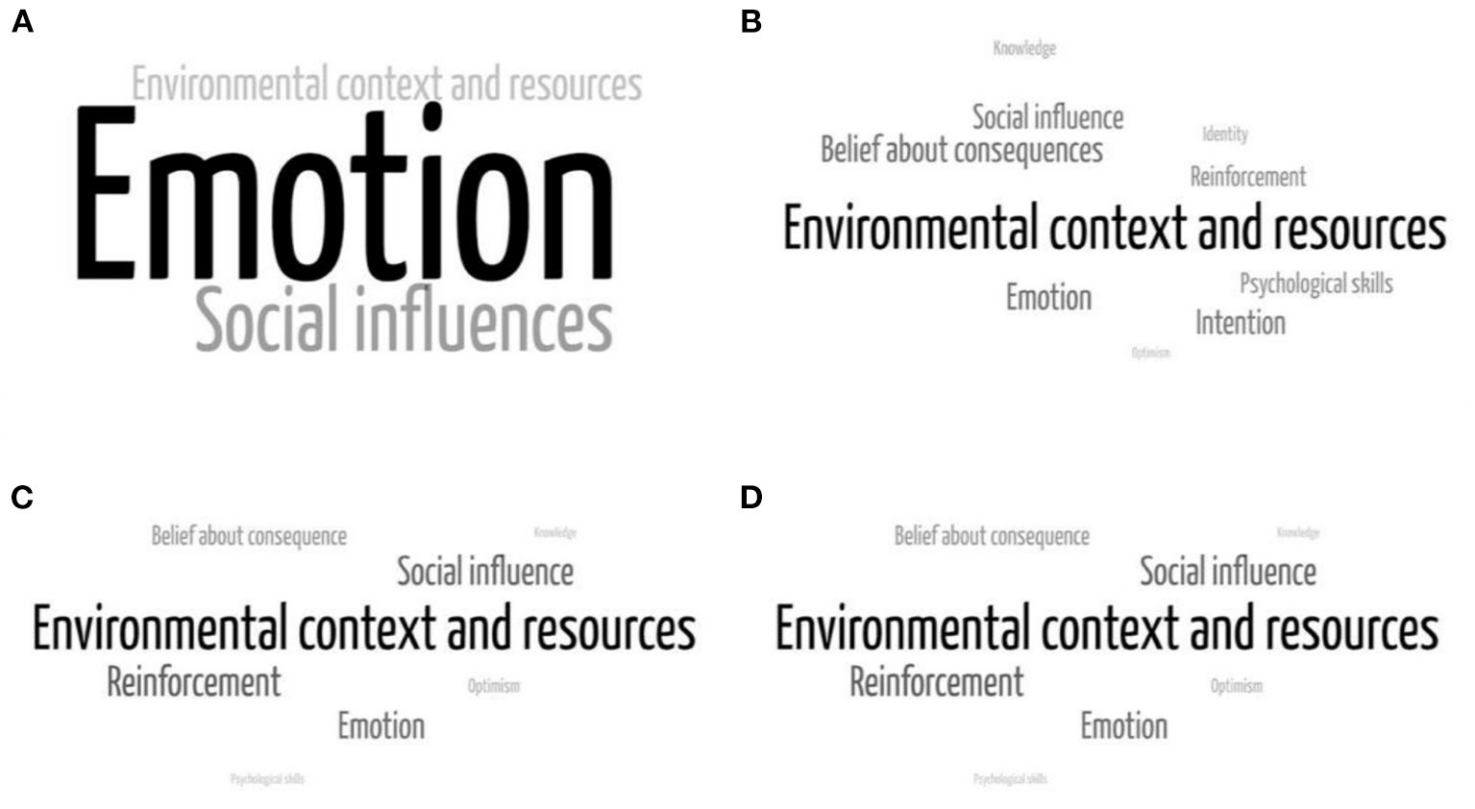
Factors influencing intercity mobility restriction: **(A)** barriers among compliers, **(B)** facilitators among compliers, **(C)** barriers among non-compliers, and **(D)** facilitators among compliers.

Concerning intercity mobility restriction, emotion, social influences, and environmental context and resources were the most commonly mentioned TDF domain, which hampers intercity mobility restriction (Figure 1). In addition to the environmental context and resource domain, compliers mentioned beliefs about consequence and emotion as their common facilitators. However, non-compliers mentioned reinforcement and social influences as their common facilitators.

Environmental context and resources domain was the most commonly mentioned factors influencing intercity mobility restriction. Job-related and transportation-related contexts could either facilitate or hinder informants from avoiding intercity mobility. On the other hand, participants perceived that they could not stay in the region if religious, family and cultural events were held in other regions during Ramadan and Eid Al-Fitr.

“As Indonesians, normally we do homecoming for doing *sungkeman* (kneeling down) tradition.” (Informant 019, M, < 45 years old, the implementer of mobility restriction).

“I have to travel between regions because my office is outside the region” (Participant 005, F, < 45 years old, non-implementer of mobility restriction).

Informants mentioned that emotions such as boredom, homesickness, loneliness, and fear of planned lockdown had hindered them to stay in their area of residence. Belief about consequences, such as believing that staying in their region would protect them from COVID-19 infection and could end the pandemic, emerged as one of the most commonly mentioned facilitators of intercity mobility restriction.

“I miss my wife since she works in Yogyakarta.” (Informant 001, M, > 45 years old, Non-implementer of mobility restriction).

“I remain staying in this city so that the pandemic will end soon.” (Informant 002, F, >45 years old, Implementer of mobility restriction).

### 3.3. Physical distancing

**Figure 2:**
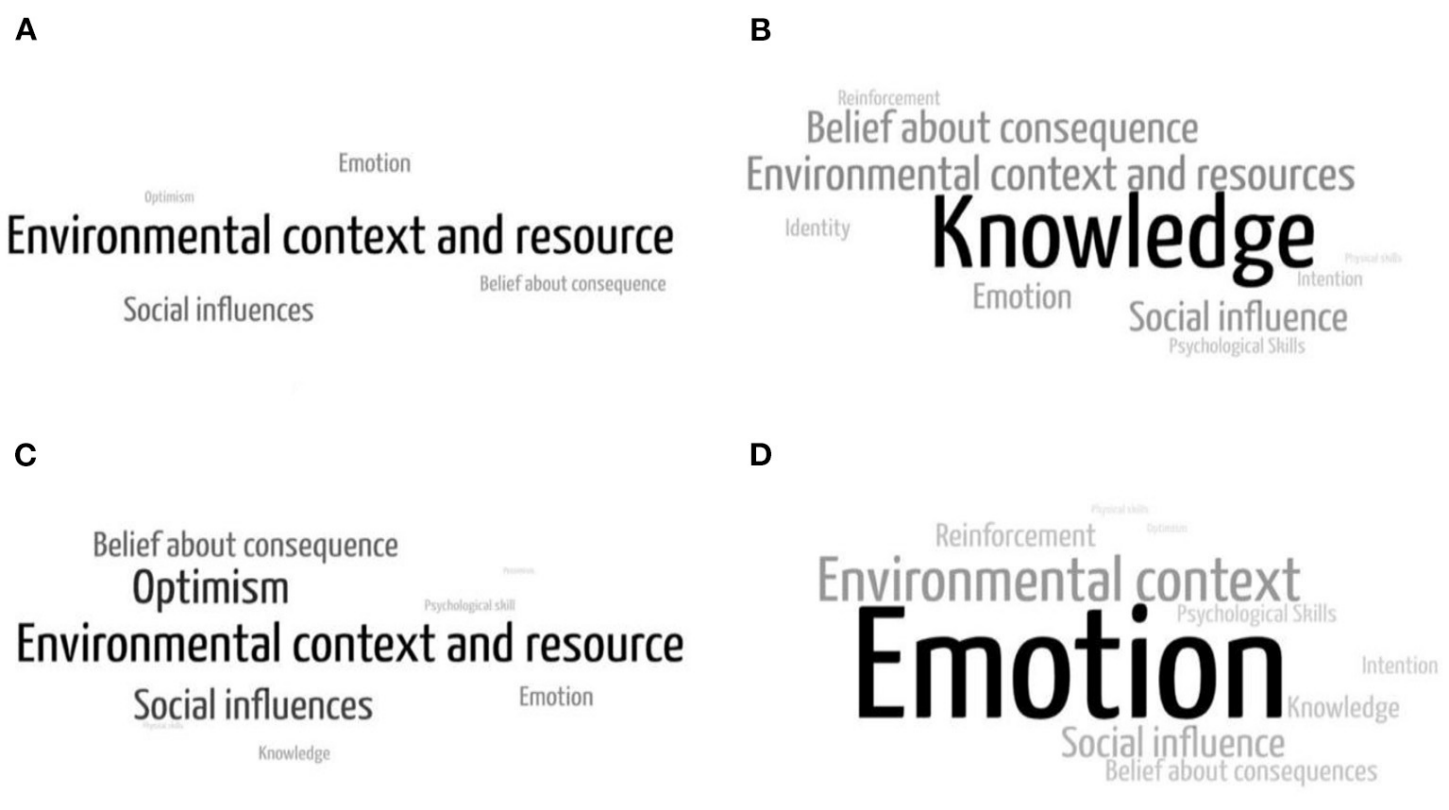
Factors influencing physical distancing: **(A)** barriers among compliers, **(B)** facilitators among compliers, **(C)** barriers among non-compliers, and **(D)** facilitators among compliers.

Among both compilers and non-compliers, environmental context and resource domain were the most commonly mentioned either as barriers or facilitators. At the same time, social influence was the most commonly mentioned barrier to physical distancing behavior (Figure 2). In addition to the environmental and social influence domain, compliers mentioned emotion, while non-compliers, on the contrary, mentioned optimism as a barrier to physical distancing. Compliers also mentioned knowledge and belief about consequences as their facilitators to physical distancing behavior, while in contrast, emotion and social influence were mentioned by non-compliers as their facilitators.

Compliers mentioned their knowledge as a facilitator of physical distancing behavior. They perceived that they could do physical distancing because they knew how to implement it, the government advice on physical distancing, and the risk of COVID-19 transmission. Furthermore, instead of knowledge related to COVID-19, compliers also mentioned that their knowledge of home exercise and productive activities during leisure time could facilitate their compliance with physical distancing measures.

“At Primary Health Care, I waited outside. When the convenience store was full, I also waited outside. I avoid such risks because I know, and I need to be able to keep my distance from other people. However, I know that each person has a different understanding.” (Informant 001, >45 years old, the implementer of physical distancing).

Within the environmental context and resources domain, informants felt that public facilities-related context influenced their compliance with physical distancing. In addition to environmental context and resources, social influences, including influence from family, colleagues, neighbors, elders, religious leaders, and health experts, and cutting in-line culture, influenced informants' compliance with physical distancing.

While in the bank, the queue has been arranged, so there is a safe distance. However, due to the many visitors, it was still impossible to perform physical distancing in the queue (Informant 007, M, < 45 years old, non-implementer of physical distancing).

During Friday prayers, the imam advised keeping the distance between the shaft. However, another imam instead asked to close the shaft during the Eid prayer (Participant 007, M, < 45 years old, non-implementer of physical distancing).

When I was at a restaurant, I couldn't keep my distance because people were crammed into the queue (Participant 013, M, < 45 years old, non-implementer of physical distancing).

Compliers mentioned feeling awkward as their barrier to physical distancing. On the other hand, non-compliers mentioned that their sense of security hindered them from physical distancing *via* interaction with a close person, asymptomatic condition, zonation, and health screening results. In addition, their optimism that COVID-19 is not dangerous was also mentioned as a barrier to physical distancing measures. While the non-compliers mentioned fear of contracting and transmitting COVID-19 as their facilitators to apply safe distance to others, the compliers mentioned that their belief in physical distancing could protect them from COVID-19 infection, end the pandemic, provide benefits for family interaction, and grant religious rewards as their facilitators to apply physical distancing.

“Physical distancing advice is in line with religious dogma which advises men to keep a distance from women and reduce unnecessary hanging out.” (Informant 045, M, >45 years old, the implementer of physical distancing).

“I try to think positively because it can increase immunity so that I can avoid the disease without being bothered by physical distancing.” (Participant 026, F, < 45 years old, non-implementer of physical distancing).

“If possible, I will do physical distancing because it can protect myself and my family” (Participant 005, F, < 45 years old, non-implementer of physical distancing).

### 3.4. Wearing a face mask

**Figure 3:**
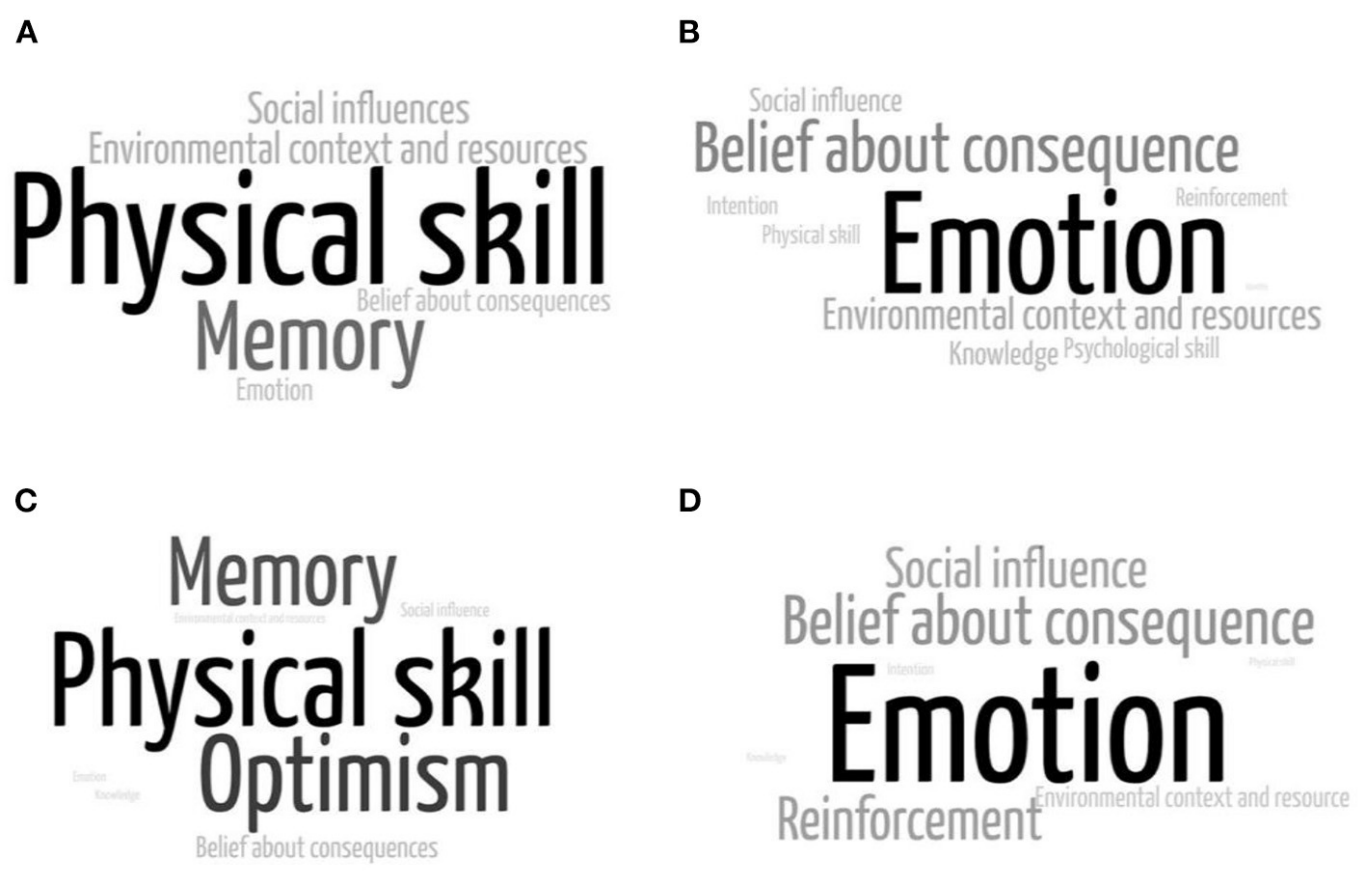
Factors influencing wearing a face mask: **(A)** barriers among compliers, **(B)** facilitators among compliers, **(C)** barriers among non-compliers, and **(D)** facilitators among compliers.

Physical skills and memory as barriers to wearing a face mask were mentioned among both compliers and non-compliers. In addition to physical skills and memory, compliers mentioned the environmental domain as their barrier to wearing a face mask. However, non-compliers mentioned optimism as their barrier to wearing a face mask (Figure 3). Among compliers, emotion, and belief about consequences as well as environmental domain were mentioned as facilitators to wearing a face mask. Meanwhile, reinforcement was mostly found as a facilitator of wearing a face mask among non-compliers.

Both compilers and non-compliers mentioned some barriers to wearing a face mask, such as being harder to breathe, having difficulty in communication, wearing dewy glasses, causing pain in the ears, and carelessness. In addition to those barriers, a job that requires clear speaking and administrative work that prohibit face mask could deter compliers from wearing a face mask. Compliers also repeatedly mentioned a sense of security caused by the asymptomatic condition or close personal interaction and their belief that COVID-19 is not dangerous as a barrier to wearing a face mask. On the other hand, non-compliers mentioned fine for a facilitator to wear a face mask. In contrast, compilers mentioned environmental factors, including supply of masks, availability of reusable masks, comfortableness of masks, mask price, provision of masks in public and workplaces, availability of alternative objects to be used as a face masks, increase of COVID-19 cases in surrounding areas, and living together with people with comorbidities as their facilitators to wear a face mask.

“Why do I have to wear a face mask? I feel healthy, so I will not carry the virus.” (Informant 013, M, < 45 years old, not adhere to wearing a face mask behavior).

“In the village, I do not need to wear a mask. They are my neighbors, so it is relatively safe.” (Informant 033, M, < 45 years old, not adhere to wearing mask behavior).

“I believe that COVID-19 is not dangerous. I do not follow government recommendations such as maintaining hand hygiene, limiting trips out of town, physical distancing, and wearing masks. My family and I have remained healthy now.” (Informant 050, M, < 45 years old, non-implementer of mobility restriction).

## 4. Discussion

Our current study investigated behavioral factors that influence three preventive measures for COVID-19, consisting of intercity mobility restriction, physical distancing, and wearing a face mask. Results from summative content analysis suggested the most commonly mentioned COM- B components and TDF domains as either facilitators or barriers to the three preventive behaviors based on compliance of the informants. The most prominent enablers and barriers to preventive behaviors can be used by practitioners and policymakers to choose to prioritize developing interventions and policies. Researchers can also use the identified determinants to further develop process models or determinant frameworks to guide practitioners in implementing preventive behaviors (52).

It could be seen that motivation factors, such as belief about consequences of the preventive behaviors, were the most frequently mentioned facilitator for either mobility restriction, physical distancing, and mask use behavior by compilers. In contrast, non-compliers were less frequently mentioned about motivation factors. This is consistent with results from other studies, which revealed that motivation for individual and community protection was the strongest facilitator of physical distancing and hand hygiene practice (31, 53). Previous studies highlighted the role of religious and community leaders in improving people's belief in the intended health behavior (54–56). In addition, informants also mentioned “social influences,” indicating the importance of advice from religious and community leaders. Therefore, involving religious and community leaders is suggested to increase people's belief about the positive consequences of mobility restriction, physical distancing, and mask-wearing behavior through persuasion and modeling (22).

On the contrary, optimism, a TDF domain categorized in the motivation component, which consisted of a sense of security and perception that COVID-19 was not dangerous, was the most frequently mentioned barrier to physical distancing and mask use behaviors among non-compliers. This is consistent with other studies which found that biased risk assessment could prevent people from sustaining preventive behaviors (57–60). It is also in accordance with the data published by Statistics Indonesia (61), which revealed that more than a quarter of Indonesian confidently felt that they would not be infected with COVID-19. The COVID-19 characteristics, which take advantage of human optimism and the increasing number of misinformation, could worsen this situation (62–64). Persuasion to increase a good understanding of the perceived threat and empowering people to take preventive behaviors for threat reduction could be suggested to reduce unrealistic optimism as barriers for physical distancing and mask-wearing (22, 65–67).

Sense of security caused by other unjustified preventive behaviors, commonly mentioned as the barrier of physical distancing, and wearing a face mask in the optimism domain, should also be taken into consideration. Informants in a previous study also perceived that their belief in other preventive behaviors as sufficient measures could hamper them from complying with physical distancing measures (68). Intervention and policy should be designed to highlight and focus more on the prioritized preventive behaviors, including physical distancing and wearing a face mask. Inattentive intervention and policy might result in people choosing their favorable behaviors and neglecting the prioritized behaviors (69–72). In addition to including messaging to communicate perceived threats and involving religious and community leaders, persuasion should also use positive framing around physical distancing and wearing a face mask to improve people's adoption of these intended behaviors (73, 74). Positively framed messaging around physical distancing and wearing a face mask could include messages that these intended behaviors could effectively protect oneself, family, and community (74, 75). The needs for clear and appropriate messaging around physical distancing and wearing a face mask are still relevant nowadays when the COVID-19 vaccination coverage in Indonesia is still low (76). While the country is still confronted with challenges in improving the COVID-19 vaccine acceptance (77), appropriate messages for the COVID-19 vaccination, physical distancing, and wearing a face mask should be considered to prevent the reduction of adherence to physical distancing and wearing a face mask (78).

The need for health promotion message nudging motivation factors could be explained by the neuroscience process of human decisions on behavior. The neo-mammal brain, especially the pre-frontal cortex (PFC), enables rationally driven behavior, and the paleo-mammal brain contributes altruistic qualities to the neocortex, including empathy, foresight, and conscience. The paleomammalian brain (limbic system) also plays a role in affective behavior, including emotions, both from the positive side (order, beliefs, and certain types of behaviors) and the negative side (need for power, egoism, intolerance, etc.). While the neo-mammalian brain maintains primitive brain functions by regulating the flow of its functions and even overriding it, the decision carried out by the neo-mammalian brain requires more complex cognitive function than the decision carried out by the paleomammalian brain, which is commonly influenced by emotional and other automatic motivation (79). In addition, our limited and incomplete information about COVID-19 could reduce the role of cognition; hence emotion dominates cognition in the process of decision-making (80).

Positively framed messaging about preventive behavior was also needed to override the influence of optimism as a barrier to preventive behavior. People tend to alter their beliefs to a greater extent in response to favorable compared with unfavorable information (81). For favorable information, stronger connectivity between the left inferior frontal gyrus (IFG) and left subcortical regions (including the amygdala, hippocampus, thalamus, putamen, and pallidum), insular cortex, is associated with greater change in belief. However, for unfavorable information, stronger connectivity between the left IFG and left pallidum, putamen, and insular cortex is associated with reduced beliefs (82).

Physical opportunities, such as job-related opportunities and economic context, emerged as barriers to mobility restriction among both compliers and non-compliers. Therefore, mobility restrictions should not be implemented in the long term since policies and interventions to modify barriers due to job-related and economic contexts were not feasible. In addition, implementing policy resulting in economic insecurity could be counterproductive to other preventive behaviors (30). While intercity travel restrictions might be useful for limiting the spread of COVID-19 in the early pandemic, other preventive behaviors such as physical distancing and mask-wearing had a bigger impact on reducing COVID-19 transmission (83). Implementation of health screening tests as a part of intercity mobility restrictions should also be evaluated. We found that some informants thought they did not need to wear a face mask and apply physical distancing since they felt a sense of security caused by the health screening test. A Cochrane systematic review also questioned the usefulness of the health screening test (84). It is because entry or exit screening without polymerase chain reaction (PCR) testing and subsequent quarantine and observation were not effectively detecting new cases to prevent transmission (84, 85).

In addition, implementing the COVID-19 screening test as a travel requirement is costly for society, and this requires national commitment and substantial additional financing. Although the fiscal, macroeconomic, and health benefits of rapid screening testing programs far exceed their costs (86), the signal value of the screening test itself is low, leading to more concerns about adherence. For some people, adherence is difficult because of living circumstances and financial-related matters. There are some concerns that low specificity (a high rate of false positives) would undercut the credibility of the screening program, reducing adherence to restrict mobility (85). Even with partial adherence to mobility restriction, low specificity would drag down the economy further by placing many health workers in isolation. Furthermore, low sensitivity allows infected individuals to fall through the cracks (85). These concerns raise questions about the public health and economic benefits of imperfect screening tests.

Overall, this study has notable strengths. First, this study draws on the triangulation metaphor and related epistemological and ontological perspectives, which determine analytic preferences and thus yield different forms of knowledge. This study shows how triangulating perspectives could extend individual-level results and how researchers should go beyond a descriptive level of analysis for convergent and inharmonious accounts to realize the potential of Multiple Perspective Interviews (MPIs). Triangulation of perspectives may be used to examine the same phenomenon from multiple perspectives and enrich understanding by allowing for new or deeper dimensions to emerge from the analysis. Second, the quality and quantity of samples in this study were collected with maximum variation (authenticity and adequacy). Third, qualitative analysis is contrasted with the COM-B model so that the findings can be meaningful for similar contexts (generalizability or resonance).

There are also several limitations to this study. The scope of the research is only Java and Madura, although economic, educational, religious, and occupational factors are representative, there are still other cultures, and differences in health facilities, technology, and infrastructure outside the two islands. The classification of compliance is only based on self-reported data; therefore, it is still subjective. Although direct observation is the best method, it is not possible to do it during the pandemic. Thus, several proxy questions were created to minimize subjectivity in this study. Respondent validations were not conducted because of the high number of informants. On the other hand, data saturation reached in 50 informants resulted in rich findings which represented our study's validity.

## 5. Conclusion

Identifying barriers and facilitators to preventive behaviors such as physical distancing and mask-wearing will be necessary for designing intervention and policy that aims to increase public adherence, especially when preparing for a future similar pandemic. Based on the most identified barriers and facilitators, persuasion and modeling involving religious leaders should be considered to improve public adherence to physical distancing and wearing a face mask. Concerning the opportunity factors that consist of the job and economic contexts, policy related to the intercity mobility restriction should be reconsidered to prevent a counterproductive result to other preventive behaviors.

## Data availability statement

The raw data supporting the conclusions of this article will be made available by the authors, without undue reservation.

## Ethics statement

The studies involving human participants were reviewed and approved by the Medical and Health Research Ethics Committee of the Faculty of Medicine, Public Health, and Nursing UGM. The patients/participants provided their written informed consent to participate in this study.

## Author contributions

RW: conceptualization, methodology, investigation, formal analysis, visualization, writing—original draft, and writing—review and editing. RH: conceptualization, investigation, project administration, funding acquisition, and writing—review and editing. AB: conceptualization, methodology, writing—review and editing, and supervision. DW: project administration, investigation, and writing—review and editing. NS: conceptualization, investigation, and writing—review and editing. PS: methodology and writing—review and editing. LA: conceptualization and methodology. MH: conceptualization and writing—review and editing. FD: methodology. All authors contributed to the article and approved the submitted version.

## References

[1] Noorimotlagh Z Jaafarzadeh N Martínez SS Mirzaee SA. A systematic review of possible airborne transmission of the COVID-19 virus (SARS-CoV-2) in the indoor air environment. Environ Res. (2021) 193:110612. 10.1016/j.envres.2020.110612 33309820 PMC7726526

[2] Rahman HS Aziz MS Hussein RH Othman HH Salih Omer SH Khalid ES. The transmission modes and sources of COVID-19: a systematic review. Int J Surg Open. (2020) 26:125–36. 10.1016/j.ijso.2020.08.017 34568614 PMC7484735

[3] Tang JW Marr LC Li Y Dancer SJ. COVID-19 has redefined airborne transmission. BMJ. (2021) 373:n913. 10.1136/bmj.n913 33853842

[4] Weber TP Stilianakis NI. Fomites, hands, and the transmission of respiratory viruses. J Occup Environ Hyg. (2021) 18:1–3. 10.1080/15459624.2020.1845343 33284735

[5] Rahimi F Abadi ATB. Implications of the emergence of a new variant of SARS-CoV-2, VUI-202012/01. Arch Med Res. (2021) 52:569–71. 10.1016/j.arcmed.2021.01.001 33526352 PMC7826083

[6] Sanyaolu A Okorie C Marinkovic A Haider N Abbasi AF Jaferi U. The emerging SARS-CoV-2 variants of concern. Ther Adv Inf Disease. (2021) 8: 20499361211024372. 10.1177/20499361211024372 34211709 PMC8216402

[7] Prem K Liu Y Russell TW Kucharski AJ Eggo RM Davies N. The effect of control strategies to reduce social mixing on outcomes of the COVID-19 epidemic in Wuhan, China: a modeling study. Lancet Public Heal. (2020) 5: e261–70. 10.1016/S2468-2667(20)30073-6 32220655 PMC7158905

[8] West R Michie S Rubin GJ Amlôt R. Applying principles of behaviour change to reduce SARS-CoV-2 transmission. Nat Hum Behav. (2020) 4:451–9. 10.1038/s41562-020-0887-9 32377018

[9] Leung K Wu JT Liu D Leung GM. First-wave COVID-19 transmissibility and severity in China outside Hubei after control measures, and second-wave scenario planning: a modelling impact assessment. Lancet. (2020) 395:1382–93. 10.1016/S0140-6736(20)30746-7 32277878 PMC7195331

[10] Zhang S Wang Z Chang R Wang H Xu C Yu X. COVID-19 containment: China provides important lessons for global response. Front Med. (2020) 14:215–9. 10.1007/s11684-020-0766-9 32212059 PMC7095399

[11] Chairil T. Indonesian government's COVID-19 measures, January-May 2020: Late response and public health securitization. J Ilmu Sos dan Ilmu Polit. (2020) 24:128–52. 10.22146/jsp.55863

[12] De Salazar P Niehus R Taylor A Buckee C Lipsitch M. Using predicted imports of 2019-nCoV cases to determine locations that may not be identifying all imported cases. medRxiv. (2020) 11:1–13. 10.1101/2020.02.04.20020495 32511458 PMC7239086

[13] Djalante R Lassa J Setiamarga D Sudjatma A Indrawan M Haryanto B. Review and analysis of current responses to COVID-19 in Indonesia: period of January to March 2020. Prog Disaster Sci. (2020) 6:100091. 10.1016/j.pdisas.2020.100091 34171011 PMC7149002

[14] Mahendradhata Y Lestari T Djalante R. Strengthening government's response to COVID-19 in Indonesia: a modified Delphi study of medical and health academics. medRxiv. (2020) 17:e20228270. 10.1101/2020.11.09.20228270 36174076 PMC9522309

[15] Chang SL Harding N Zachreson C Cliff OM Prokopenko M. Modelling transmission and control of the COVID-19 pandemic in Australia. Nat Commun. (2020) 11:5710. 10.1038/s41467-020-19393-6 33177507 PMC7659014

[16] Ngonghala CN Iboi E Eikenberry S Scotch M MacIntyre CR Bonds MH. Mathematical assessment of the impact of non-pharmaceutical interventions on curtailing the 2019 novel Coronavirus. Math Biosci. (2020) 325:108364. 10.1016/j.mbs.2020.108364 32360770 PMC7252217

[17] Satuan Tugas Penanganan COVID-19. (2020). Available online at: https://covid19.go.id/p/berita/infografis-covid-19-26-mei-2020 (accessed November 11, 2020).

[18] CNN Indonesia,. (2020). Available online at: https://www.cnnindonesia.com/nasional/20210520062939-20-644516/kasus-prokes-dan-kecelakaan-masa-lebaran-2021-naik-100-persen (accessed November 12, 2020).

[19] Satuan, Tugas Penanganan COVID-19. https://covid19.go.id/monitoring-kepatuhan-protokol-kesehatan (accessed November 12, 2020).

[20] Satuan Tugas Penanganan COVID-19. (2022). Available online at: https://covid19.go.id/artikel/2022/01/16/analisis-data-covid-19-indonesia-update-9-januari-2022 (accessed January 9, 2022).

[21] Craig P Dieppe P Macintyre S Michie S Nazareth I Petticrew M. Developing and evaluating complex interventions: the new Medical Research Council guidance. BMJ. (2008) 337:a1655. 10.1136/bmj.a1655 18824488 PMC2769032

[22] Michie S Atkins L West R. The Behaviour Change Wheel: A Guide To Designing Interventions. New York, NY: Silverback Publishing (2014).

[23] Baker R Camosso-Stefinovic J Gillies C Shaw EJ Cheater F Flottorp S. Tailored interventions to address determinants of practice. Cochrane Database Syst Rev. (2015) 4:5470. 10.1002/14651858.CD005470.pub3 25923419 PMC7271646

[24] Proctor EK Powell BJ Baumann AA Hamilton AM Santens RL. Writing implementation research grant proposals: ten key ingredients. Implement Sci. (2012) 7:1–13. 10.1186/1748-5908-7-96 23062065 PMC3541090

[25] Connell LA McMahon NE Tyson SF Watkins CL Eng JJ. Mechanisms of action of an implementation intervention in stroke rehabilitation: a qualitative interview study. BMC Health Serv Res. (2016) 16:534. 10.1186/s12913-016-1793-8 27716247 PMC5045623

[26] Courtenay M Rowbotham S Lim R Peters S Yates K Chater A. Examining influences on antibiotic prescribing by nurse and pharmacist prescribers: a qualitative study using the theoretical domains framework and COM-B. BMJ Open. (2019) 9:177. 10.1136/bmjopen-2019-029177 31221892 PMC6588983

[27] Ellis K Pears S Sutton S. Behavioural analysis of postnatal physical activity in the UK according to the COM-B model: a multi-methods study. BMJ Open. (2019) 9:682. 10.1136/bmjopen-2018-028682 31377705 PMC6686995

[28] Fulton E Brown K Kwah K Wild S. StopApp: using the Behaviour Change Wheel to develop an app to increase uptake and attendance at NHS Stop Smoking Services. Healthcare. (2016) 4:31. 10.3390/healthcare4020031 27417619 PMC4934584

[29] Handley MA Harleman E Gonzalez-Mendez E Stotland NE Althavale P Fisher L. Applying the COM-B model to creation of an IT-enabled health coaching and resource linkage program for low-income Latina moms with recent gestational diabetes: The STAR MAMA program. Implement Sci. (2016) 11:1–15. 10.1186/s13012-016-0426-2 27193580 PMC4870786

[30] Ojo SO Bailey DP Hewson DJ Chater AM. Perceived barriers and facilitators to breaking up sitting time among desk-based office workers: a qualitative investigation using the TDF and COM-B. Int J Environ Res Public Health. (2019) 16:903. 10.3390/ijerph16162903 31416112 PMC6720704

[31] Coroiu A Moran C Campbell T Geller AC. Barriers and facilitators of adherence to social distancing recommendations during COVID-19 among a large international sample of adults. Capraro V, editor PLoS One. (2020) 15:e0239795. 10.1371/journal.pone.0239795 33027281 PMC7540845

[32] Shin SH Ji H Lim H. Heterogeneity in preventive behaviors during COVID-19: Health risk, economic insecurity, and slanted information. Soc Sci Med. (2021) 278:113944. 10.1016/j.socscimed.2021.113944 33940436

[33] Benham JL Lang R Burns KK MacKean G Léveillé T McCormack B. Attitudes, current behaviours and barriers to public health measures that reduce COVID-19 transmission: a qualitative study to inform public health messaging. PLoS ONE. (2021) 16:1–14. 10.1371/journal.pone.0246941 33606782 PMC7895406

[34] Cristancho S Goldszmidt M Lingard L Watling C. Qualitative research essentials for medical education. Singapore Med J. (2018) 59:622–7. 10.11622/smedj.2018093 30009321 PMC6301871

[35] O'Brien BC Harris IB Beckman TJ Reed DA Cook DA. Standards for reporting qualitative research: a synthesis of recommendations. Acad Med. (2014) 89:1245–51. 10.1097/ACM.0000000000000388 24979285

[36] Braun V Clarke V. Successful Qualitative Research in Psychology: A Practical Guide for Beginners. London: SAGE. (2013).

[37] World Health Organization - Indonesia. https://cdn.who.int/media/docs/default-source/searo/indonesia/who-situation-report-2.pdf?sfvrsn=fc754e00_2 (accessed November 13, 2020).

[38] Olivia S Gibson J Nasrudin R. Indonesia in the time of COVID-19. Bull Indones Econ Stud. (2020) 56:143–74. 10.1080/00074918.2020.1798581

[39] Tong A Sainsbury P Craig J. Consolidated criteria for reporting qualitative research (COREQ): a 32-item checklist for interviews and focus groups. Int J Qual Heal Care. (2007) 19:349–57. 10.1093/intqhc/mzm042 17872937

[40] Kidd PS Parshall MB. Getting the focus and the group: Enhancing analytical rigor in focus group research. Qual Health Res. (2000) 10:293–308. 10.1177/104973200129118453 10947477

[41] Leung FH Savithiri R. Spotlight on focus groups. Can Fam Physician. (2009) 55:218–9. 19221085 PMC2642503

[42] DeJonckheere M Vaughn LM. Semistructured interviewing in primary care research: a balance of relationship and rigour. Family Med Commun Health. (2019) 7:e000057. 10.1136/fmch-2018-000057 32148704 PMC6910737

[43] Braun V Clarke V. Using thematic analysis in psychology. Qual Res Psychol. (2006) 3:77–101. 10.1191/1478088706qp063oa

[44] Boyatzis R. Transforming Qualitative Information. London: SAGE Publications. (1998).

[45] Hsieh HF Shannon SE. Three approaches to qualitative content analysis. Qual Health Res. (2005) 15:1277–88. 10.1177/1049732305276687 16204405

[46] Jackson C Eliasson L Barber N Weinman J. Applying COM-B to medication adherence: a suggested framework for research and interventions. Bull Eur Heal Psychol Soc. (2014) 16:7–17.

[47] Barker F Atkins L Lusignan de. Applying the COM-B behaviour model and behaviour change wheel to develop an intervention to improve hearing-aid use in adult auditory rehabilitation. Int J Audiol. (2016) 55:S90–8. 10.3109/14992027.2015.1120894 27420547

[48] Hartarto RB Wardani DTK Azizurrohman M. A Qualitative study of conditional cash transfer and education aspirations: evidence from Yogyakarta. J Soc Serv Res. (2021) 47:1–10. 10.1080/01488376.2021.1918314

[49] Bussiere AE Patey AM Francis JJ Sales AE Grimshaw JM Canada PRIme Plus Team. Identifying factors likely to influence compliance with diagnostic imaging guideline recommendations for spine disorders among chiropractors in North America: a focus group study using the theoretical domains framework. Implement Sci. (2012) 7:2–11. 10.1186/1748-5908-7-82 22938135 PMC3444898

[50] Cane J O'Connor D Michie S. Validation of the theoretical framework. Implement Sci. (2012) 7:37. 10.1186/1748-5908-7-37 22530986 PMC3483008

[51] Lake AJ Browne JL Rees G Speight J. What factors influence uptake of retinal screening among young adults with type 2 diabetes: a qualitative study informed by the theoretical domains framework. J Diabetes Complications. (2017) 31:997–1006. 10.1016/j.jdiacomp.2017.02.020 28363730

[52] Nilsen P. Making sense of implementation theories, models and frameworks. Impl Sci. (2015) 53:1–13. 10.1186/s13012-015-0242-0 25895742 PMC4406164

[53] Gibson Miller J Hartman TK Levita L Martinez AP Mason L McBride O. Capability, opportunity, and motivation to enact hygienic practices in the early stages of the COVID-19 outbreak in the United Kingdom. Br J Health Psychol. (2020) 25:856–64. 10.1111/bjhp.12426 32415918 PMC7276910

[54] Padmawati RS Heywood A Sitaresmi MN Atthobari J MacIntyre CR Soenarto Y. Religious and community leaders' acceptance of rotavirus vaccine introduction in Yogyakarta, Indonesia: a qualitative study. BMC Public Health. (2019) 19:368. 10.1186/s12889-019-6706-4 30943929 PMC6446267

[55] Syiroj ATR Pardosi JF Heywood AE. Exploring parents' reasons for incomplete childhood immunisation in Indonesia. Vaccine. (2019) 37:6486–93. 10.1016/j.vaccine.2019.08.081 31522808

[56] Byron MJ Cohen JE Gittelsohn J Frattaroli S Nuryunawati R Jernigan DH. Influence of religious organisations' statements on compliance with a smoke-free law in Bogor, Indonesia: a qualitative study. BMJ Open. (2015) 5:8111 10.1136/bmjopen-2015-008111 26667011 PMC4679994

[57] Floyd DL Prentice-Dunn S Rogers RW. A meta-analysis of research on protection motivation theory. J Appl Soc Psychol. (2000) 30:407–29. 10.1111/j.1559-1816.2000.tb02323.x

[58] Fragkaki I Maciejewski DF Weijman EL Feltes J Cima M. Human responses to COVID-19: the role of optimism bias, perceived severity, and anxiety. Pers Individ Dif. (2020) 176:110781. 10.1016/j.paid.2021.110781 33623178 PMC7891080

[59] Kuzmanovic B Rigoux L. Valence-dependent belief updating: computational validation. Front Psychol. (2017) 8:1–11. 10.3389/fpsyg.2017.01087 28706499 PMC5489622

[60] Sharot T Korn CW Dolan RJ. How unrealistic optimism is maintained in the face of reality. Nat Neurosci. (2011) 14:1475–9. 10.1038/nn.2949 21983684 PMC3204264

[61] Badan Pusat Statistik. Perilaku Masyarakat di Masa Pandemi COVID-19. Indonesia: Badan Pusat Statistik (2020).

[62] Bottemanne H Morlaàs O Fossati P Schmidt L. Does the Coronavirus epidemic take advantage of human optimism bias? Front Psychol. (2020) 11:1–5. 10.3389/fpsyg.2020.02001 32982839 PMC7479219

[63] Laato S Islam AKMN Islam MN Whelan E. What drives unverified information sharing and cyberchondria during the COVID-19 pandemic? Eur J Inf Syst. (2020) 29:288–305. 10.1080/0960085X.2020.1770632

[64] Romer D Jamieson KH. Conspiracy theories as barriers to controlling the spread of COVID-19 in the U.S. Soc Sci Med. (2020) 263:113356. 10.1016/j.socscimed.2020.113356 32967786 PMC7502362

[65] Dowd JB Andriano L Brazel DM Rotondi V Block P Ding X. Demographic science aids in understanding the spread and fatality rates of COVID-19. Proc Natl Acad Sci U S A. (2020) 117:9696–8. 10.1073/pnas.2004911117 32300018 PMC7211934

[66] Pearce JM Lindekilde L Parker D Rogers MB. Communicating with the public about marauding terrorist firearms attacks: results from a survey experiment on factors influencing intention to “Run, Hide, Tell” in the United Kingdom and Denmark. Risk Anal. (2019) 39:1675–94. 10.1111/risa.13301 30893483

[67] Peters GJY Ruiter RAC Kok G. Threatening communication: a critical re-analysis and a revised meta-analytic test of fear appeal theory. Health Psychol Rev. (2013) 7:8–31. 10.1080/17437199.2012.703527 23772231 PMC3678850

[68] Moore RC Lee A Hancock JT Halley M Linos E. Experience with social distancing rarly in the COVID-19 pandemic in the United States: implications for public health messaging. medRxiv. (2020). 10.1101/2020.04.08.20057067 32511643 PMC7276999

[69] Best K. Study of COVID-19 Risk Communication Finds ‘Optimistic Bias' Slows Preventive Behavior - UConn Today: UConn Communications. (2020). Available online at: https://today.uconn.edu/2020/04/study-covid-19-risk-communication-finds-optimistic-bias-slows-preventive-behavior/# (accessed November 14, 2020).

[70] Hedlund J. Haddon Memorial Lecture. Inj Prev. (2000) 1:82–9.

[71] Luckman A Zeitoun H Isoni A Loomes G Vlaev I Powdthavee N. Risk compensation during COVID-19: the impact of face mask usage on social distancing. Osf. (2020) 355:722. 10.31219/osf.io/rb8he 35073133

[72] Mantzari E Rubin GJ Marteau TM. Is risk compensation threatening public health in the COVID-19 pandemic? BMJ. (2020) 370:m2913. 10.1136/bmj.m2913 32713835

[73] Gallagher KM Updegraff JA. Health message framing effects on attitudes, intentions, and behavior: a meta-analytic review. Ann Behav Med. (2012) 43:101–16. 10.1007/s12160-011-9308-7 21993844

[74] Michie S West R Rogers MB Bonell C Rubin GJ Amlôt R. Reducing SARS-CoV-2 transmission in the UK: a behavioural science approach to identifying options for increasing adherence to social distancing and shielding vulnerable people. Br J Health Psychol. (2020) 25:945–56. 10.1111/bjhp.12428 32428385 PMC7276722

[75] Koon AD Mendenhall E Eich L Adams A Borus ZA. A spectrum of (Dis)Belief: Coronavirus frames in a rural midwestern town in the United States. Soc Sci Med. (2021) 272:113743. 10.1016/j.socscimed.2021.113743 33592395 PMC8723978

[76] Sutarsa N. Indonesia's Vaccine Campaign Hits Speed Bumps | East Asia Forum. East Asia Forum. (2021). Available online at: https://www.eastasiaforum.org/2021/05/15/indonesias-vaccine-campaign-hits-speed-bumps/ (accessed June 29, 2021).

[77] Harapan H Wagner AL Yufika A Winardi W Anwar S Gan AK. Acceptance of a COVID-19 vaccine in Southeast Asia: a cross-sectional study in Indonesia. Front Publ Health. (2020) 8:381. 10.3389/fpubh.2020.00381 32760691 PMC7372105

[78] Trogen B Caplan A. Risk compensation and COVID-19 vaccines. Ann Intern Med. (2021) 174:858–9. 10.7326/M20-8251 33646837 PMC7983310

[79] Keyes CD. Ethical judgment and brain function: an interpretation of Paul D. MacLean's hypothesis J Soc Evol Syst. (1992) 15:387–98. 10.1016/1061-7361(92)90025-9

[80] Luo J Yu R. Follow the heart or the head: The interactive influence model of emotion and cognition. Front Psychol. (2015) 6:1–14. 10.3389/fpsyg.2015.00573 25999889 PMC4422030

[81] Eil D Rao JM. The Good News-Bad News effect: asymmetric processing of objective information about yourself. Am Econ J Microeconomics. (2011) 3:114–38. 10.1257/mic.3.2.114

[82] Moutsiana C Charpentier CJ Garrett N Cohen MX Sharot T. Human frontal–subcortical circuit and asymmetric belief updating. J Neurosci. (2015) 35:14077–85. 10.1523/JNEUROSCI.1120-15.2015 26490851 PMC4683678

[83] Quilty BJ Diamond C Liu Y Gibbs H Russell TW Jarvis CI. The effect of travel restrictions on the geographical spread of COVID-19 between large cities in China: a modelling study. BMC Med. (2020) 18:1–10. 10.1186/s12916-020-01712-9 32814572 PMC7437104

[84] Burns J Movsisyan A Stratil JM Coenen M Emmert-Fees KM Geffert K. Travel- related control measures to contain the COVID-19 pandemic: a rapid review. Cochrane Database Syst Rev. (2020) 28:717. 10.1002/14651858.CD013717 33502002

[85] Pettengill MA McAdam AJ. Can we test our way out of the COVID-19 pandemic? J Clin Microbiol. (2020) 58:1–5. 10.1128/JCM.02225-20 32843531 PMC7587108

[86] Atkeson A Droste M Mina M Stock J. Economic Benefits of COVID-19 Screening Tests. Cambridge, MA: Cambridge University Press (2020).

