# Supplementary material for: Facilitators and barriers of preventive behaviors against COVID-19 during Ramadan: A phenomenology of Indonesian adults
Source: Front Public Health. 2023 Mar 21;11:960500. doi: 10.3389/fpubh.2023.960500 (PMC10073479; doi:10.3389/fpubh.2023.960500)
Supplement: Supplementary file 1 [file Table_1.DOCX]

Supplemental File 1. Summary of Theoretical Domain Framework Domain

|  | Barriers | | | | Facilitators | | | |
| --- | --- | --- | --- | --- | --- | --- | --- | --- |
|  | TDF domain | Frequency of mentions | | Percentage of mentions | TDF domain | Frequency of mentions | | Percentage of mentions |
| Intercity mobility restriction | | | | | | | | |
| Compliers | Emotion | 6 | 86% | | Environmental context and resources | 20 | 34% | |
|  | Social influences | 1 | 13% | | Belief about consequences | 7 | 12% | |
|  | Environmental context and resources | 1 | 13% | | Emotion | 7 | 12% | |
|  |  |  |  | | Intention | 6 | 10% | |
|  |  |  |  |  | Social influence | 6 | 10% | |
|  |  |  |  |  | Psychological skills | 4 | 7% | |
|  |  |  |  |  | Reinforcement | 4 | 7% | |
|  |  |  |  |  | Identity | 2 | 3% | |
|  |  |  |  |  | Knowledge | 2 | 3% | |
|  |  |  |  |  | Optimism | 1 | 2% | |
| People who did not comply | Environmental context and resources | 57 | 50% | | Environmental context and resources | 22 | 39% | |
|  | Emotion | 27 | 23% | | Reinforcement | 10 | 18% | |
|  | Social influences | 13 | 11% | | Social influence | 9 | 16% | |
|  | Optimism | 10 | 9% | | Emotion | 8 | 14% | |
|  | Reinforcement | 2 | 2% | | Belief about consequence | 4 | 7% | |
|  | Identity | 3 | 3% | | Optimism | 2 | 4% | |
|  | Knowledge | 3 | 3% | | Knowledge | 1 | 2% | |
|  |  |  |  | | Psychological skills | 1 | 2% | |
| Physical distancing | | | | | | | | |
| People who complied | Environmental context and resource | 13 | | 54% | Knowledge | 18 | | 22% |
|  | Social influences | 5 | | 21% | Environmental context and resources | 16 | | 19% |
|  | Emotion | 3 | | 13% | Belief about consequence | 14 | | 17% |
|  | Belief about consequence | 2 | | 8% | Social influence | 12 | | 14% |
|  | Optimism | 1 | | 4% | Emotion | 9 | | 11% |
|  |  |  | |  | Identity | 4 | | 5% |
|  |  |  |  |  | Psychological Skills | 3 | | 4% |
|  |  |  |  |  | Reinforcement | 3 | | 4% |
|  |  |  |  |  | Intention | 3 | | 4% |
|  |  |  |  |  | Physical skills | 1 | | 1% |
| People who did not comply | Environmental context and resource | 41 | | 30% | Emotion | 27 | | 29% |
|  | Optimism | 32 | | 24% | Environmental context | 26 | | 28% |
|  | Social influences | 25 | | 19% | Social influence | 12 | | 13% |
|  | Belief about consequence | 17 | | 13% | Reinforcement | 7 | | 7% |
|  | Emotion | 10 | | 7% | Belief about consequences | 6 | | 6% |
|  | Knowledge | 5 | | 4% | Knowledge | 6 | | 6% |
|  | Psychological skill | 3 | | 2% | Intention | 4 | | 4% |
|  | Pessimism | 1 | | 1% | Psychological Skills | 4 | | 4% |
|  | Physical skills | 1 | | 1% | Optimism | 1 | | 1% |
|  |  |  | |  | Physical skills | 1 | | 1% |
| Wearing a face mask | | | | | | | | |
| People who complied | Physical skill | 21 | | 60% | Emotion | 30 | | 21% |
|  | Memory | 8 | | 23% | Belief about consequence | 30 | | 21% |
|  | Environmental context and resources | 2 | | 6% | Environmental context and resources | 24 | | 17% |
|  | Social influences | 2 | | 6% | Knowledge | 13 | | 9% |
|  | Belief about consequences | 1 | | 3% | Social influence | 13 | | 9% |
|  | Emotion | 1 | | 3% | Psychological skill | 10 | | 7% |
|  |  |  | |  | Intention | 8 | | 6% |
|  |  |  |  |  | Reinforcement | 8 | | 6% |
|  |  |  |  |  | Physical skill | 8 | | 6% |
|  |  |  |  |  | Identity | 1 | | 1% |
| People who did not comply | Physical skill | 12 | |  | Emotion | 11 | | 26% |
|  | Optimism | 8 | |  | Belief about consequence | 9 | | 21% |
|  | Memory | 7 | |  | Reinforcement | 7 | | 16% |
|  | Belief about consequences | 5 | |  | Social influence | 6 | | 14% |
|  | Social influence | 2 | |  | Environmental context and resource | 6 | | 14% |
|  | Emotion | 1 | |  | Intention | 2 | | 5% |
|  | Knowledge | 1 | |  | Physical skill | 1 | | 2% |
|  | Environmental context and resources | 1 | |  | Knowledge | 1 | | 2% |
