# Supplementary material for: Facilitators and barriers of preventive behaviors against COVID-19 during Ramadan: A phenomenology of Indonesian adults
Source: Front Public Health. 2023 Mar 21;11:960500. doi: 10.3389/fpubh.2023.960500 (PMC10073479; doi:10.3389/fpubh.2023.960500)
Supplement: Supplementary file 2 [file Table_2.DOCX]

Supplemental File 2. Themes, Subthemes, and Quotes

| COM-B component | TDF domain | Sub-theme | Sample quotes |
| --- | --- | --- | --- |
| *Interregional Mobility restriction* | | | |
| Psychological Capability | Psychological Skills | The ability to do online shopping | I do not think of any difficulty (to stay in this city). I can buy food, vegetables, clothes, and other necessities online. (Participant 022, F, <45 years old, implementer of mobility restriction) |
|  | Knowledge | Knowledge of government advice on preventive behaviours | At that time, I did not know if the government forbade it (intercity mobility restriction). After knowing the health protocol, I no longer do it. (Participant 024, M, >45 years old, non-implementer of mobility restriction) |
|  |  | Knowledge about risk of COVID-19 transmission | I understand that when I use public facilities during the trip, I run the risk of being infected so I could become an asymptomatic person. It might be dangerous had I have met my parents. (Participant 035, F, <45 years old, implementer of mobility restriction) |
|  |  | Knowledge to do video calling with family | Even though I miss them, I know that I am still able to make video calls with my family. (Participant 019, M, <45 years old, implementer of mobility restriction) |
|  |  | Knowledge to do activities during leisure time | When I have not been assigned to job tasks, I did not know what to do. Therefore, I decided to go for homecoming. (Participant 032, F, <45 years old, non-implementer of mobility restriction) |
|  |  |  | I was not bored when I stayed in this city because I could do several hobbies during leisure time such as painting. (Participant 035, F, <45 years old, implementer of mobility restriction) |
| Physical opportunity | Environmental context and resource | The workplace system allows work from home | I went homecoming to Jogja since my workplace allowed me to work from home. (Participant 001, M, > 45 years old, Non-implementer of mobility restriction) |
|  |  |  | Alhamdulillah all work can be done online. Meetings are held online. Giving lectures can also be done online. I can still do work from home without having to go to my office at Yogyakarta. (Participant 034, M, <45 years old, implementer of mobility restriction) |
|  |  | Job availability | I went for homecoming because I got laid off. (Participant 003, F, <45 years old, non-implementer of mobility restriction) |
|  |  |  | I have to travel between regions because my office is outside the region. (Participant 005, F, <45 years old, non-implementer of mobility restriction) |
|  |  |  | I still have a job in Jakarta so I prefer to stay here. (Participant 007, M, <45 years old, non-implementer of mobility restriction) |
|  |  | Workload | At that time, my workload was so high that I could not go for homecoming. (Participant 011, M, <45 years old, non-implementer of mobility restriction) |
|  |  | Workplace tasks | I had to go outside from the region because there were office files that must be managed in West Java. (Participant 007, M, <45 years old, non-implementer of mobility restriction) |
|  |  |  | I did not get any office tasks in another city so I could stay in this city. (Participant 016, M, <45 years old, implementer of mobility restriction) |
|  |  | Regional policies requiring self-quarantine for travellers | There is a self-quarantine policy for travellers so I decided to just stay here. (Participant 001, M, > 45 years old, Non-implementer of mobility restriction) |
|  |  | Availability of inter-region public transportation | At that period, I had difficulty of finding public transportation to go to another region. Thus, I decided to postpone my homecoming. (Participant 003, F, <45 years old, non-implementer of mobility restriction) |
|  |  | Availability of private vehicle | I could do intercity travelling because I had a private vehicle. (Participant 008, M, <45 years old, non-implementer of mobility restriction) |
|  |  | Limitation of intracity public transportation | If I stayed in this city, I had trouble in finding public transportation or online transportation then I decided to go for homecoming. (Participant 007, M, <45 years old, non-implementer of mobility restriction) |
|  |  | Administrative requirements for inter-region travellers | While traveling to the other regions, I encountered several difficulties such as medical screening examination and required documents. (Participant 004, M, >45 years old, Non-implementer of physical distancing) |
|  |  | Number of COVID-19 cases in the region | I went out of Jakarta because the number of cases was very large and I predicted that the number would increase sharply. (Participant 006, F, >45 years old, non-implementer of mobility restriction) |
|  |  |  | I found it easier to live here and not to go to other regions because the number of cases here was still low. (Participant 020, F, <45 years old, non-implementer of mobility restriction) |
|  |  | Ignorant people | In my neighbourhood, many people are ignorant. I could be in danger because of them. Therefore, I went to Jogja. (Participant 006, F, >45 years old, non-implementer of mobility restriction) |
|  |  | Religious events | There was a family event that made me have to go to Central Java. My wife passed away one year ago so I had to hold a *tahlilan* (repeated recitation) event. (Participant 007, M, <45 years old, non-implementer of mobility restriction) |
|  |  | Family events | I went out of town because I had to attend a brother's wedding. (Participant 020, M, <45 years old, non-implementer of mobility restriction) |
|  |  | Cultural events | As Indonesians, normally we do homecoming for doing *sungkeman* *(*kneeling down*)* tradition. (Participant 019, M, <45 years old, implementer of mobility restriction) |
|  |  | Internet access | I could access the internet more adequately for online classes in my hometown, hence i decided to go for homecoming. (Participant 046, M, <45 years old, non-implementer of mobility restriction) |
|  |  | Access to staple goods | I thought that staying in this region is hard since I found it difficult to eat as the opening hours of food stall are limited. (Participant 011, M, <45 years old, non-implementer of mobility restriction) |
|  |  |  | I can easily buy groceries because there is a supermarket within the apartment complex. (Participant 022, F, <45 years old, implementer of mobility restriction) |
|  |  | Availability of social assistance funds | I do not receive social assistance funds. How can I survive without it? (Participant 010, M, <45 years old, non-implementer of mobility restriction) |
|  |  | Availability of online shopping facilities | I did not find any reason to go outside this city because I could shop online and almost all of the stores in this city provide online shopping facilities. (Participant 002, F, <45 years old, implementer of mobility restriction) |
|  |  | Living with people with comorbidity or high-risk people | I do not want to go out of town because I have old parents. (Participant 017, F, >45 years old, implementer of mobility restriction) |
| Social opportunity | Social influence | Influence from family, neighbours, and colleague | My family actually told me to go for homecoming. (Participant 003, F, <45 years old, non-implementer of mobility restriction) |
|  |  |  | My office mates support me not to go for homecoming and we still communicate with each other since we do not go for homecoming... My family also understand if I do not go for homecoming. (Participant 002, F, >45 years old, implementer of mobility restriction) |
|  |  |  | My family was actually afraid that I would come for homecoming. However, I assured them that I would stick to health protocols...My co-workers forbid me from traveling between regions and warned me about the risk of contracting during the trip (Participant 007, M, <45 years old, non-implementer of mobility restriction) |
|  |  |  | Neighbours forbade me to go for homecoming. (Participant 023, M, >45 years old, non-implementer of mobility restriction) |
|  |  | Leadership | My leader always stressed that we must be a concerned society by following the rules of the government to stay in this city. (participant 019, M, <45 years old, implementer of mobility restriction) |
| Reflective motivation | Optimism | Sense of security caused by the zonation | I came from Serang which was not a red zone, so I believed that it is safe to go to other regions. (Participant 009, F, <45 years old, non-implementer of mobility restriction) |
|  |  | Sense of security caused by other preventive behaviours | I feel safe when I travel because I wear a mask and wash my hands frequently. (Participant 015, M, <45 years old, non-implementer of mobility restriction) |
|  |  | Sense of security caused by health preparedness | I believe that the health facilities here are better prepared than those in other areas. (Participant 022, F, <45 years old, implementer of mobility restriction) |
|  |  | Sense of security caused by asymptomatic condition | I am sure that I do not have any symptoms resembling COVID-19 such as cough and fever, so I ventured out on a trip to visit my family (Participant 037, F, <45 years old, non-implementer of mobility restriction) |
|  |  | Belief that COVID-19 is not dangerous | I believe that COVID-19 is not dangerous. I do not follow government recommendations such as maintaining hand hygiene, limiting trips out of town, physical distancing and wearing masks. My family and I have remained healthy now. (Participant 050, M, <45 years old, non-implementer of mobility restriction) |
|  | Belief about consequence | Belief that restricting mobility will protect from COVID-19 | By staying in Jakarta, I feel safe from the risk of transmission while in public transportation. (Participant 014, F, <45 years old, implementer of mobility restriction) |
|  |  | Belief that adherence to health protocols will immediately end of the pandemic | I remain staying in this city so that the pandemic will end soon. (Participant 002, F, >45 years old, Implementer of mobility restriction) |
|  |  | Belief that travellers will stigmatised as sources of COVID-19 transmission | I am afraid that my colleagues will stigmatise me as the source of infection after I returned from homecoming. (Participant 009, F, <45 years old, non-implementer of mobility restriction) |
|  | Intention | Intention | I must follow the government's advice. They already consisted of experts who have found the best solution for us. (Participant 045, M, <45 years old, implementer of mobility restriction) |
|  | Identity | Role model | As a community leader, I should be a role model for society. (Participant 029, M, >45 years old, implementer of mobility restriction) |
|  |  | Devoted child | As a son, I have to take care of my mom, so I decided to go for homecoming. (Participant 025, M, <45 years old, non-implementer of mobility restriction) |
| Automatic motivation | Reinforcement | Punishment from workplace | If I stayed in this city, i would not get a punishment from my institution since I am a civil servant. (Participant 002, F, >45 years old, Implementer of mobility restriction) |
|  |  | Administrative requirements for inter-region travellers | I find it difficult to complete the administrative requirements for intercity travelling. (participant 019, M, <45 years old, implementer of mobility restriction) |
|  | Emotion | Fear of contracting COVID-19 | I was so scared of contracting COVID-19 during travelling, so I immediately washed all the things I brought when I boarded the train. (Participant 022, F, <45 years old, non-implementer of mobility restriction) |
|  |  | Boredom | My family is bored, so we want to travel out of town to Yogyakarta (Participant 050, M, <45 years old, non-implementer of mobility restriction) |
|  |  | Fear of transmitting COVID-19 | When I went for homecoming, actually I was afraid of transmitting COVID-19 to people in my hometown. (Participant 003, F, <45 years old, non-implementer of mobility restriction) |
|  |  | Fear of planned lockdown | I am afraid that the access out of city will be closed, so I went for homecoming before it happened. (Participant 018, M, <45 years old, non-implementer of mobility restriction) |
|  |  | Homesick | I miss my wife since she works in Yogyakarta. (Participant 001, M, > 45 years old, Non-implementer of mobility restriction) |
|  |  |  | The only barrier to stay here is that I miss my husband. I have not met him yet in the last four months. (Participant 002, F, >45 years old, Implementer of mobility restriction) |
|  |  | Loneliness | I would feel alone during Eid if I did not go home. (Participant 008, F, <45 years old, Non-implementer of mobility restriction) |
| Physical distancing | | | |
| Psychological Capability | Knowledge | Knowledge and skill to do physical distancing | At Primary Health Care, I waited outside. When the convenience store was full, I also waited outside. I avoid such risks because I know and I need to be able to keep my distance from other people. However, I know that each person has different understanding. (Participant 001, >45 years old, implementer of physical distancing) |
|  |  | Knowledge of health impact of COVID-19 | I strictly complies to physical distancing because I know that older people are the most vulnerable one. (Participant 001, M, >45 years old, Implementer of physical distancing) |
|  |  | Knowledge about risk of COVID-19 transmission | I comply with physical distancing because I know that the more the crowd and the more people we met, the higher the potential for spreading the virus. We also never know who is infected with the virus and who is not infected. (Participant 002, F, >45 years old, Implementer of physical distancing) |
|  |  | Knowledge of government advice on preventive behaviours | I do not know anything about the advice from the government to apply physical distancing. (Participant 004, M, >45 years old, Non-implementer of physical distancing) |
|  |  |  | I think that the government is advising to return to normal life now so I do not have to apply physical distancing anymore. (Participant 028, M, >45 years old, non-implementer of physical distancing behaviour) |
|  |  | Knowledge and skills of home exercise | I perform exercise every morning. I can do this at home because I know that there are exercise videos that can be played on my mobile phone and I can follow them. (Participant 001, M, >45 years old, Implementer of physical distancing) |
|  |  | Knowledge and skills to do productive activities at home | I can stay at home so I do not have to meet other people because I can do productive activities at home such as making crafts. (Participant 025, M, <45 years old, non-implementer of physical distancing) |
|  | Psyhological skill | Ability to adapt with physical distancing norm | Now, I understand that keeping a safe distance while speaking with others is a new norm. (Participant 001, M, >45 yars old, implementer of physical distancing) |
|  |  | Interpersonal skills to ask people to do physical distancing | When I meet people who think that they do not have to apply physical distancing, I remind them. (Participant 024, M, >45 years old, non-implementer of physical distancing) |
|  |  |  | I wanted to remind them but I could not. (Participant 029, M, >45 years old, non-implementer of physical distancing) |
|  |  | the ability to do online shopping | Luckily, I was able to contact the groceries sellers via WhatsApp so that I could buy vegetables and other foods online. Thus, I can avoid crowded in the market. (Participant 028, M, >46 years old, non-implementer of physical distancing) |
| Physical capability | Physical capability | Having comorbidities | Since I am already old, I have to comply with health protocols, including physical distancing. (Participant 006, F, >45 years old, Non-implementer of physical distancing) |
|  |  | Physical condition needing services that don't allow physical distancing | I felt achy and needed a massage by a therapist which made it impossible to keep a safe distance. (Participant 017, F, >45 years old, non-implementer of physical distancing) |
|  |  | Difficulty in communication | I had difficulty in communication while doing physical distancing, so I had to speak louder. (Participant 017, F, >45 years old, non-implementer of physical distancing) |
| Environmental opportunity | Environmental context and resources | Availability of public places allowing physical distancing | While in the bank, the queue has actually been arranged, so there is a safe distance. However, due to a large number of visitors still, it was impossible to perform physical distancing in queue. (Participant 007, M, <45 years old, non-implementer of physical distancing) |
|  |  |  | When I eat at the food stall, of course, I cannot apply physical distancing since it is narrow and crowded so that visitors are jostling while eating. (Participant 013, M, <45 years old, non-implementer of physical distancing) |
|  |  | Availability of transportation facilities allowing physical distancing | I went to the office by taking the pick-up vehicle provided by the office, which was only filled with 50% of passengers. (Participant 002, F, >45 years old, Implementer of physical distancing) |
|  |  |  | When I got on the plane, there was someone sitting just beside me coughing aloud. I could not do anything, so I just gave up. (Participants 006, F, <45 years old, non-implementer of physical distancing) |
|  |  | Availability of occupational environments allowing physical distancing | In order to allow physical distancing at the office, we work in alternated schedule. (Participant 024, M, >45 years old, non-implementer of physical distancing) |
|  |  | Availability of alternative places to shop for basic necessities | Sometimes, I shop at a neighbour's shop to avoid the crowded market. (Participants 006, F, <45 years old, non-implementer of physical distancing) |
|  |  | Logistic support during self-quarantine | As long as there are food assistance and entertainment facilities, self-quarantine for 15 to 16 days, there will not be a problem. (Participant 025, M, <45 years old, non-implementer of physical distancing) |
|  |  | Family, cultural, religious, and social event | It is difficult to apply for physical distancing. Especially during the *tahlilan* (repeated recitation) event when we have to sit close to each other. (Participant 023, M, >45 years old, non-implementer of physical distancing) |
|  |  |  | There are no Friday prayers in my office so I do not get involved in religious activities and it becomes possible to apply physical distancing. (Participant 008, M, <45 years old, non-implementer of physical distancing) |
|  |  |  | At that time, I had to guard the social assistance distribution activities that were visited by the community in large numbers so that these activities made it difficult for me to manage the situation. (Participant 016, M, <45 years old, non-implementer of physical distancing) |
|  |  | Living with people with comorbidity or high-risk people | I have to strict with physical distancing measures since I live with my father who suffered from diabetes and my mother who suffered from hypertension. (Participant 006, F, >45 years old, Non-implementer of physical distancing) |
|  |  | Workload | I have no workload, so I can stay at home all the time. (Participant 006, F, >45 years old, Non-implementer of physical distancing) |
|  |  | Job type | My job requires me to be in contact with patients so that it is not possible to do physical distancing. |
|  |  |  | Of course, I cannot keep my distance from the passengers because the length of the motorbike seat is shorter than one metre. |
|  |  | Ignorant people | I have been trying to keep my distance from others. However, if other people were ignorant, it would still be hard for me to keep my distance from others. (Participant 003, F, <45 years old, implementer of physical distancing) |
|  |  | Availability of quarantine facilities | During the self-quarantine period, my family had a separate room for me so that I could be separated from other family members. (Participant 008, M, <45 years old, non-implementer of physical distancing) |
|  |  | Availability of private vehicle | I do not have a private vehicle, so I am very dependent on public transportation even though driving public transportation requires more efforts to implement physical distancing. (Participant 002, F, >45 years old, Implementer of physical distancing) |
|  |  |  | I can drive my own car so I can avoid crowds on public transport. (Participant 043, F, <45 years old, implementer of physical distancing) |
|  |  | Physical distancing signage | In the mosque, there is a marker that makes it easy to apply physical distancing during prayer. (Participant 029, M, >45 years old, non-implementer of physical distancing) |
|  |  | Having children | I have two children. When going out with them, of course, it is hard to apply physical distancing. Small children cannot understand, especially when they meet their coevals. (Participant 022, F, <45 years old, implementer of physical distancing) |
|  |  | Presence and Increase of positive cases in surrounding area | After a case was found in my area, I tried to apply physical distancing more frequently. (Participant 024, M, >45 years old, non-implementer of physical distancing) |
|  |  |  | Compared to Yogyakarta, the number of cases here is totally higher. Therefore, I am implementing more stringent health protocols. (Participant 034, M, <45 years old, implementer of physical distancing) |
| Social opportunity | Social influence | Norm in social interaction | The public have already understood that we must maintain our distance while having interaction during pandemic. (Participant 003, F, <45 years old, implementer of physical distancing) |
|  |  |  | Handshake and keeping close to each other during the talk is a norm, so it will be hard to keep one-metre distance with other people. (Participant 007, M, <45 years old, non-implementer of physical distancing) |
|  |  | Cutting in line norm | At work, I cannot keep my distance because everyone is in hurry and no one is in line. (Participant 004, M, >45 years old, non-implementer of physical distancing) |
|  |  |  | When I was in the restaurant, I couldn’tt keep my distance because people were crammed into the queue. (Participant 013, M, <45 years old, non-implementer of physical distancing) |
|  |  | Influence from family, colleagues, and neighbourhood to adhere with physical distancing behaviour | Neighbours remind each other via Whatsapp group to comply with physical distancing behaviour. (Participant 001, M, >45 years old, implementer of physical distancing) |
|  |  |  | Sometimes, there are relatives who complain if I implement physical distancing when they visit my house. (Participant 002, F, >45 years old, Implementer of physical distancing) |
|  |  | Influence from elders | Many elders say that I am excessive if I reject to do handshaking. (Participant 006, F, >45 years old, non-implementer of physical distancing) |
|  |  | Influence from religious leaders | During Friday prayers, the imam advised keeping the distance between the *shaf* (prayer row). However, another imam instead asked to fill the *shaf* during the Eid prayer... During the sermon, the imam reminded us not to do handshakes. (Participant 007, M, <45 years old, non-implementer of physical distancing) |
|  |  | Influence from health experts | I got information from my customers who work in the health department. He said that COVID-19 is only a conspiracy, so there is no need to be afraid. Health experts have spoken like that. (Participant 013, M, <45 years old, non-implementer of physical distancing) |
|  |  |  | My friends who work as health workers advising to apply physical distancing to avoid COVID-19. (Participant 023, M, >45 years old, non-implementer of physical distancing) |
| Reflective motivation | Belief about consequence | Belief that adherence to health protocols will immediately end the pandemic | If everyone adheres to health protocols, this pandemic will end soon, economic will recover, and family can gather again. (Participant 001, M, >45 years old, implementer of physical distancing) |
|  |  | Belief that physical distancing will protect from COVID-19 | If possible, I will do physical distancing because it can protect myself and my family (Participant 005, F, <45 years old, non-implementer of physical distancing) |
|  |  | Belief that travellers will be stigmatised as a source of COVID-19 transmission | I was worried that I would be seen as the source of the virus, so I stayed at home. I only went out when I was drying clothes. (Participant 006, F, >45 years old, non-implementer of physical distancing) |
|  |  | Belief that physical distancing will be stigmatised as an excessive behaviour | If we meet people who are ignorant, we are said to be excessive. (Participant 002, F, >45 years old, implementer of physical distancing) |
|  |  | Belief that people who do physical distancing will be stigmatised as the source of infection | If I apply physical distancing, people will think that I am a virus carrier. (Participant 018, M, <45 years old, non-implementer of physical distancing) |
|  |  | Belief about the economic consequence | If I appear to be doing physical distancing, I am afraid that people will not buy my items. (Participant 015, M, <45 years old, non-implementer of physical distancing) |
|  |  | Beliefs about the Consequences of religion | I believe that there should be no distance between people during worship. (Participant 018, M, <45 years old, non-implementer of physical distancing) |
|  |  |  | Physical distancing advice is in line with religious dogma which advises men to keep a distance from women and reduce unnecessary hanging out. (Participant 045, M, >45 years old, implementer of physical distancing) |
|  |  | Belief about the family consequence | I have more time to interact with my family. (Participant 017, F, >45 years old, non-implementer of physical distancing) |
|  |  | Belief about mental health consequence | I am afraid to become stressed if I reduce my interactions with other people. (Participant 017, F, >45 years old, non-implementer of physical distancing) |
|  | Identity | Responsibility towards family | I have to take responsibility if my family gets infected. (Participant 001, M, >45 years old, Implementer of physical distancing) |
|  |  | Role model | I am afraid that people will follow me if I break the rules of physical distancing. (Participant 019, M, <45 years old, implementer of physical distancing) |
|  | Optimism | Sense of security caused by other preventive behaviours | Activities can run normally because they are safe. There are friends who have helped disinfect the mosque. (Participant 024, M, >45 years old, non-implementer of physical distancing) |
|  |  |  | I try to think positively because it can increase immunity so that I can avoid the disease without being bothered by physical distancing. (Participant 026, F, <45 years old, non-implementer of physical distancing) |
|  |  |  | God willing, it is safe because I do a lot of physical activities until I sweat and I get exposed to the sun. I am also not afraid to increase my immunity... I also drink herbs. (Participant 028, M, <45 years old, non-implementer of physical distancing behaviour) |
|  |  | Sense of security caused by close person interaction (office mates, neighbours & family) | I still do not implement physical distancing during the funeral event since they are my family. (Participant 032, F, <45 years old, non-implementer of physical distancing) |
|  |  |  | When I attend a cultural event in my area, I do not need to keep my distance because they are my neighbours whom I have known, so it is relatively safe. (Participant 033, M, <45 years old, non-implementer of physical distancing) |
|  |  | Sense of security caused by asymptomatic condition | Besides, they are healthy people so I do not have to apply physical distancing from them (Participant 008, M, <45 years old, non-implementer of physical distancing) |
|  |  | Sense of security caused by the zonation | I do not implement physical distancing because I am in a safe zone area. (Participant 047, F, <45 years old, non-implementer of physical distancing) |
|  |  | Sense of security caused by the result of rapid diagnostic test | I had checked myself with a rapid test and the result was non-reactive. Therefore, I became calm and did not need to do self-quarantine. (Participant 039, M, <45 years old, non-implementer of physical distancing) |
|  |  | Belief that COVID-19 is not dangerous | I believe that COVID-19 is not dangerous. I do not follow government recommendations such as maintaining hand hygiene, limiting trips out of town, physical distancing and wearing masks. My family and I have remained healthy now. (Participant 050, M, <45 years old, non-implementer of mobility restriction) |
|  | Pessimism | Pessimism that physical distancing will not protect against COVID-19 | Even if we try to stay away from the disease, we will be exposed if that is our destiny. For example, the viral news of a mother who did not go anywhere, yet still infected with COVID-19. (Participant 033, M, <45 years old, non-implementer of physical distancing) |
|  | Intention | Intention to obey the government advice | I just want to follow the protocol from the government. (Participant 011, M, <45 years old, implementer of physical distancing) |
| Automatic motivation | Emosion | Fear of contracting COVID-19 | I have to keep my distance despite the crowded situation since I am afraid of catching the disease. (Participant 001, M, >45 years old, implementer of physical distancing) |
|  |  |  | I was afraid of catching it when I was in the crowded market, but I still have to shop for daily necessities. (Participant 005, F, <45 years old, Non-implementer of physical distancing) |
|  |  | Fear of transmitting COVID-19 | I do not want my parents to be contracted by the virus because of me. Therefore, I try to stay at home all the time. (Participant 006, F, >45 years old, Non-implementer of physical distancing) |
|  |  | Feeling awkward | We used to do handshakes and kisses on the cheeks, now we are getting awkward if we do not do it. (Participant 003, F, <45 years old, non-implementer of physical distancing) |
|  | Reinforcement | Sanction/punishment for health protocol violators | In my neighbourhood, there are sanctions for breaking health protocols so I do not want to break them. (Participant 003, F, <45 years old, implementer of physical distancing) |
| Wearing a face mask | | | |
| Psychological Capability | Knowledge | Knowledge about COVID-19 transmission | I know that this disease is transmitted through sprinkled saliva. If I use a mask, I can reduce my risk of getting splashed. (Participant 009, F, <45 years old, adhere to wearing mask behaviour) |
|  |  | Knowledge of government advice on preventive behaviours | After knowing that the government is intensifying the use of masks, I always wear a mask. (Participant 048, F, <45 years old, adhere to wearing mask behaviour) |
|  |  | Knowledge to reuse the mask | I am happy to know how to use reusable cloth masks. However, I still need to pay attention on how to wash it and the limits of its use. (Participant 007, M, <45 years old, not adhere to wearing mask behaviour) |
|  |  | Knowledge to find other alternatives to face mask | When I forget to bring my mask, I know an alternative object that can be used to cover my nose and mouth. (Participant 017, F, >45 years old, adhere to wearing a mask behaviour) |
|  | Memory | Carelessness | I forgot to bring a mask when I was going to ride a motorbike taxi. (Participant 007, M, <45 years old, not adhere to wearing a mask behaviour) |
|  | Psychological skills | The ability to deal with stigma from others | No problem. I am not ashamed of what other people say if I wear a face mask. (Participant 024, M, >45 years old, adhere to wearing mask behaviour) |
|  |  | Ability to make wearing masks as a habit | Before the pandemic, I already worn a mask when riding a motorcycle. (Participant 014, F, <45 years old, adherence to wearing a mask behaviour) |
| Physical capability | Physical skill | Hard to breathe | When I put on the mask, I felt short of breath. (Participant 003, F, <45 years old, implementer of wearing mask behaviour) |
|  |  | Ability to adapt with breathing difficulty | Over time, I became more and more adapted to wearing a mask so I did not get short of breath anymore. (Participant 001, M, >45 yars old, adhere to wearing mask behaviour) |
|  |  | Difficulty in communication | When I was teaching, it was difficult for me to speak clearly when I was wearing a mask. (Participant 002, F, <45 years old, adhere to wearing mask behaviour) |
|  |  | Dewy eyeglasses | I am an eyeglasses wearer. Wearing a mask will cause dewy in my eyeglasses. (Participant 032, F, <45 years old, not adhere to wearing mask behaviour) |
|  |  | Having comorbidities | I always wear a mask because I have asthma which can worsen the symptoms of COVID-19. (Participant 029, M, >45 years old, adhere to wearing mask behaviour) |
|  |  | Pain on ears | When I wear the mask for too long, I feel pain on my ears. (Participant 039, M, <45 years old, not adhere to wearing mask behaviour) |
| Environmental opportunity | Environmental resource and context | Mask supply | If the supply of masks in the office runs out, I still have a personal supply of cloth masks. (Participant 009, F, <45 years old, adhere to wearing mask behaviour) |
|  |  |  | I ran out of stock of masks because all my masks were still wet. Okay, I just left the house without a mask. (Participant 012, F, <45 years old, non-adhere to wearing mask behaviour) |
|  |  | Availability of reusable mask | I have a mask that can be washed if it gets dirty so I do not have any problems. (Participant 015, M, <45 years old, non-adhere to wearing mask behaviour) |
|  |  | Availability of comfortable mask | I chose a mask that is comfortable to wear and does not cause shortness of breath (Participant 011, M, <45 years old, adhere to wearing mask behaviour) |
|  |  | Mask price | I had a hard time at the start of the pandemic because the masks were very expensive. (Participant 006, F, >45 years old, adhere to wearing mask behaviour) |
|  |  | Provision of masks in occupational and public places | For people who forget to bring a mask, there are masks available for use at the place for Eid prayer. My workplace also provides hand sanitisers and masks. (Participant 007, M, <45 years old, not adhere to wearing mask behaviour) |
|  |  | Other alternative objects that can be used as a mask | If I forget to bring my mask, I use a handkerchief that I often carry them in my bag. (Participant 007, M, <45 years old, not adhere to wearing mask behaviour) |
|  |  | Presence and Increase of positive cases in surrounding area | In the past, people here were not disciplined in wearing masks. However, they became disciplined after there were cases in our area. I also became more disciplined. (Participant 024, M, >45 years old, adhere to wearing mask behaviour) |
|  |  | Living with people with comorbidity or high-risk people | I live with my parents who are at high risk, so I have to take more care of myself. (Participant 006, F, >45 years old, adhere to wearing mask behaviour) |
|  |  | Job type | I work as a trainer which requires me to speak clearly. (Participant 002, F, <45 years old, adhere to wearing mask behaviour) |
|  |  | Administrative requirements that do not allow wearing masks | In some places, a face scan is required to enter so you have to remove the mask. (Participant 022, F, <45 years old, adhere to wearing mask behaviour) |
| Social opportunity | Social support | Support from family, neighbour, colleagues, and health care providers | My family supports each other in wearing masks. In our area, neighbours also support each other to wear masks. (Participant 001, M, >45 years old, adhere to wearing mask behaviour) |
|  | Social pressure | Social pressure | If I do not wear a mask, I may suffer from a social pressure. (Participant 007, M, <45 years old, not adhere to wearing mask behaviour) |
|  | Modelling | Modelling from the local people | The neighbours around me were not wearing masks, hence I followed them not to wear a mask. (Participant 004, M, >45 years old, non-adhere to wearing a mask behaviour) |
|  | Social norm | Norm in social interaction | Maybe someone is influenced. However, Alhamdulillah, people around me have understood that interaction still has to keep a distance and wear a mask, then do not shake hands. We have already taken this as commonplace. (Participant 034, M, <45 years old, implementer of physical distancing) |
| Reflective motivation | Identity | Role model | If I obey wearing a mask, hopefully, others will follow. (Participant 001, M, >45 years old, adhere to wearing mask behaviour) |
|  |  | Responsibility towards family | I am responsible for my family's health. (Participant 003, F, <45 years old, implementer of wearing mask behaviour) |
|  | Belief about consequence | Belief that adherence to health protocols will immediately end of the pandemic | If we ignore government programs, how long this pandemic will be. On the other hand, if we follow the government's program, I hope this pandemic can end soon and life returns to normal. (Participant 007, M, <45 years old, not adhere to wearing mask behaviour) |
|  |  | Belief that wearing a mask will protect from COVID-19 | By wearing a mask, I can protect myself and my family. (Participant 005, F, <45 years old, adhere to wearing mask behaviour) |
|  |  | Belief that wearing a mask will protect from pollution | If I wear a mask, I can avoid air pollution so that I can breathe fresh air. (Participant 010, M, <45 years old, non-adhere to wearing a face mask behaviour) |
|  |  | Beliefs about the consequences of religion | If we take care of ourselves, we can take care of others too. It will be rewarded (by God) ... By wearing a mask, it is easier for my intention to wear the veil according to religious advice. (F) (Participant 006, F, >45 years old, adhere to wearing mask behaviour) |
|  |  |  | If I come back for the mask, I will be late for Friday prayers. (Participant 039, M, <45 years old, not adhere to wearing mask behaviour) |
|  |  | Belief about social consequences | If I wear a mask, people will suspect that I am sick or I have the virus. (Participant 018, M, <45 years old, not adhere to wearing mask behaviour) |
|  |  |  | When I wear a mask, some people say, "You are overreacting. Why should wear a mask?" (Participant 039, M, <45 years old, not adhere to wearing mask behaviour) |
|  |  | Belief about economic consequences | If I use a mask, I am afraid that my income will decrease because there will be customers who turn to other agents. (Participant 007, M, <45 years old, not adhere to wearing mask behaviour) |
|  |  | Belief about health consequences beyond the COVID-19 | By wearing a mask, I also avoided the common cold. Before wearing a mask, I often get colds. (Participant 024, M, >45 years old, adhere to wearing mask behaviour) |
|  |  |  | If I do not use a mask, I am afraid my skin will turn dark. (Participant 031, M, <45 years old, adhere to wearing mask behaviour) |
|  | Optimism | Sense of security caused by asymptomatic condition | Why I have to wear a face mask? I feel healthy, so I will not carry the virus. (Participant 013, M, <45 years old, not adhere to wearing a face mask behaviour) |
|  |  | Sense of security caused by close person interaction (office mates, neighbours & family) | In the village, I do not need to wear a mask. They are my neighbors, so it is relatively safe. (Participant 033, M, <45 years old, not adhere to wearing mask behaviour) |
|  |  | Belief that COVID-19 is not dangerous | I believe that COVID-19 is not dangerous. I do not follow government recommendations such as maintaining hand hygiene, limiting trips out of town, physical distancing and wearing masks. My family and I have remained healthy now. (Participant 050, M, <45 years old, non-implementer of mobility restriction) |
|  | Intention | Intention to obey the government advice | Because the government recommended it, I did it. (Participant 016, M, <45 years old, adhere to wearing mask behaviour) |
| Automatic motivation | Emotion | Fear of contracting COVID-19 | ...I am also afraid of contracting the disease, so I always wear a face mask (Participant 002, F, <45 years old, adhere to wearing mask behaviour) |
|  |  | Fear of transmitting COVID-19 | I am afraid if I do not wear a mask, I will transmit the disease. (Participant 002, F, <45 years old, adhere to wearing mask behaviour) |
|  |  | Feeling awkward | When I wear a mask during chat, I still feel awkward. (Participant 036, F, <45 years old, adhere to wearing mask behaviour) |
|  | Reinforcement | Sanction/punishment for health protocol violators in occupational and public places | If I do not wear a mask, I cannot enter public places. (Participant 010, M, <45 years old, not adhere to wearing mask behaviour) |
|  |  |  | My office requires wearing a mask (Participant 025, M, <45 years old, adhere to wearing mask behaviour) |
